# African Swine Fever and Its Epidemiological Course in Lithuanian Wild Boar

**DOI:** 10.3390/v13071276

**Published:** 2021-06-30

**Authors:** Katja Schulz, Marius Masiulis, Christoph Staubach, Alvydas Malakauskas, Gediminas Pridotkas, Franz J. Conraths, Carola Sauter-Louis

**Affiliations:** 1 Friedrich-Loeffler-Institut, Federal Research Institute for Animal Health, Institute of Epidemiology, Südufer 10, 17493 Greifswald-Insel Riems, Germany; Christoph.Staubach@fli.de (C.S.); Franz.Conraths@fli.de (F.J.C.); Carola.Sauter-Louis@fli.de (C.S.-L.); 2 Emergency Response Division, State Food and Veterinary Service, Siesiku 19, LT-07170 Vilnius, Lithuania; marius.masiulis@vmvt.lt (M.M.); Alvydas.Malakauskas@lsmuni.lt (A.M.); 3 Dr. L. Kriauceliunas Small Animal Clinic, Veterinary Academy, Lithuanian University of Health Sciences, Tilzes Street 18, LT-47181 Kaunas, Lithuania; 4 National Food and Veterinary Risk Assessment Institute, J. Kairiūkščio Street 10, LT-08409 Vilnius, Lithuania; gediminas.pridotkas@nmvrvi.lt; 5 Department of Veterinary Pathobiology, Veterinary Academy, Lithuanian University of Health Sciences, Tilzes Street 18, LT-47181 Kaunas, Lithuania

**Keywords:** disease control, surveillance, epidemiological course, prevalence, population density

## Abstract

African swine fever (ASF) has been present in Lithuania since 2014. It is mainly the wild boar population that is affected. Currently, little is known about the epidemiological course of ASF in Lithuania. In the present study, ASF surveillance data from 2016–2021 were analyzed. The numbers of samples taken from hunted wild boar and wild boar found dead per year and month were recorded and the prevalence was estimated for each study month and administrative unit. A Bayesian space–time model was used to calculate the temporal trend of the prevalence estimates. In addition, population data were analyzed on a yearly basis. Most samples were investigated in 2016 and 2017 and originated from hunted animals. Prevalence estimates of ASF virus-positive wild boar decreased from May 2019 onwards. Seroprevalence estimates showed a slight decrease at the same time, but they increased again at the end of the study period. A significant decrease in the population density was observed over time. The results of the study show that ASF is still present in the Lithuanian wild boar population. A joint interdisciplinary effort is needed to identify weaknesses in the control of ASF in Lithuania and to combat the disease more successfully.

## 1. Introduction

African swine fever (ASF) was first identified in 1921 [1]. In the following decades, it mainly affected the African continent, but after the first introduction into Portugal in 1957, several countries in Europe also experienced outbreaks caused by ASF virus genotype I [2]. The disease was almost completely eliminated from the European continent in 1995; only Sardinia remained endemic until recently [3,4]. In 2007, ASF virus of genotype II was introduced into Georgia, presumably through contaminated waste on a ship arriving from East Africa [5]. Since then, the disease has spread widely, now affecting a large number of countries—not only in Europe, but also in Asia [6,7,8]. In 2014, the new ASF epidemic also hit the European Union (EU) when the disease was introduced from the east, most probably Belarus. Lithuania was the first affected EU member state [9,10]. Shortly afterwards, but still in the course of the year 2014, the first ASF cases emerged in Poland, Latvia and Estonia [11]. In the following years, further EU member states became affected, including Belgium, the Czech Republic and Germany [12,13,14]. Except for Romania, where outbreaks in domestic pig holdings dominate [15], the disease is mainly carried by the wild boar population in most affected countries [16,17]. The involvement of wild boar makes the disease extremely difficult to control [18,19,20], particularly if ASF is introduced by wild boar migrating across borders, as happened in the Baltic states, Poland and recently in Germany [12,21,22]. By contrast, disease elimination has been possible in Belgium and in the Czech Republic, where ASF was introduced only at a single point in time and space, presumably by human activities [14,23]. It is widely known that ASF can be transmitted from domestic pigs to wild boar and vice versa. It is important to note that countries where only the wild boar population is infected suffer from international trade restrictions, even if the domestic pig population if free from ASF [24]. Thus, control of ASF in wild boar and ultimately its elimination is crucial to protect the pig industry, to maintain global trade and to secure the economy of affected countries.

Since ASF reached Lithuania in 2014, great efforts have been made to combat the disease. Intensive disease surveillance and control measures were implemented and hunting management was adapted. Biosecurity in the context of hunting was improved and strengthened. Hunting was intensified and incentives were paid for targeted hunting of adult and sub-adult females, for reporting wild boar found dead and for disposing the carcasses safely by burial. Despite these control measures, the disease is still present in the wild boar population and has now spread throughout the country [9]. Only in the west of the country are small parts (less than 2000 km^2^) still free from ASF. In addition, outbreaks have occurred in domestic pig holdings every year since 2014 [10,25]. Recent data analyses in Estonia and Latvia suggest that the efforts to fight ASF are rewarded, since the number of ASF virus (ASFV)-positive wild boar has decreased. In contrast, the number of hunted wild boar that tested positive for ASFV-specific antibodies, but negative for ASFV, has increased [24,26,27,28]. This course of disease may result from an accumulation of surviving animals and the simultaneous absence of new infections [27,28]. The circulation of an attenuated virus or the existence of ASFV carrier animals has also been discussed as possible causes for this epidemiological course of ASF [26,29,30,31]. In Lithuania, however, the analyses of ASF surveillance data performed until 2018 indicate an ongoing epidemic situation with regular occurrence of newly infected wild boar [9,25,26].

The epidemiology of ASF in wild boar and the drivers for persistence, transmission and spread are still not fully understood. Thus, the options for controlling the disease reliably and effectively are limited and constantly under debate. The recent introduction of ASF into the German wild boar population, its further spread and the ongoing epidemic in Poland and Lithuania emphasize the unabated need for further research and thus for a continuing effort to close knowledge gaps. In an attempt to support these efforts, we analyzed comprehensive ASF surveillance and wild boar population data from Lithuania. By including the most recent data and advanced analyses, we aimed at complementing and completing existing research results.

## 2. Materials and Methods

### 2.1. Surveillance Data

Lithuanian ASF wild boar surveillance data were obtained from the CSF/ASF wild boar surveillance database of the European Union [32]. The relevant authorities approved the use of the data. Each data record corresponds to the surveillance data for a single wild boar and contains information about place (county, municipality and the smallest administrative unit (the eldership)), where the animal was found or shot, the time (day/month/year) of sampling, age (<1 year and >1 years), sex of the wild boar and the origin of sample. The “origin” refers to how it was recorded: whether the sample was taken from an apparently healthy hunted wild boar (active surveillance) or from a wild boar found dead, shot because it was sick or killed in a road traffic accident (RTA) (passive surveillance). Furthermore, the data of the laboratory test results were recorded.

Laboratory investigations were conducted at the National Food and Veterinary Risk Assessment Institute, which is the National Reference Laboratory for ASF in Lithuania. The laboratory methods and sampling procedure were deployed as previously described [9,10].

Data from 2 January 2016 to 12 April 2021 (64 study months) at the municipality level were used. Data records before 2016 were excluded because they were often incomplete; in particular, geographical information was lacking. For each year, the number of samples examined per km^2^ was calculated for each municipality.

Samples were mainly recorded as originating from hunted wild boar and wild boar found dead. Only 32 samples (recorded in 2016 and 2017) originated from wild boar shot, because they were sick. No wild boar samples were recorded that originated from wild boar killed in RTAs. Data records from wild boar shot sick were excluded in all analyses to avoid inconsistencies. In addition, all data records lacking age information were excluded (*n* = 2965). Figures illustrating the numbers of samples from hunted wild boar and wild boar found dead per year were created using the packages “ggplot2” and “cowplot” in the software R (https://www.r-project.org/, accessed on 29 June 2021). All maps were generated using the software ArcGIS ArcMap 10.8.1 (ESRI, Redlands, CA, USA).

### 2.2. Prevalence Estimates

ASFV prevalence estimates were calculated for hunted wild boar and for wild boar found dead separately. To this end, the number of hunted wild boar that had tested positive for ASFV genome, irrespective of the serological test result, was divided by the total number of hunted wild boar with a conclusive ASFV genome detection result. The same was done for wild boar found dead. Furthermore, seroprevalence estimates were calculated for apparently healthy hunted wild boar that had tested positive for ASFV-specific antibodies and were negative for ASFV genome by PCR. Thus, the number of wild boar with the above result were divided by the total number of wild boar that had been tested serologically and virologically and had a conclusive test result in both assays.

Prevalence estimates were determined for each study month and for each of the 54 affected municipalities. Yearly prevalence estimates were calculated for each of the two age classes. These prevalence estimates were tested for differences using the non-parametric Kruskal–Wallis test. A *p*-value < 0.05 was considered as statistically significant. All prevalence estimations, statistical tests and confidence intervals were calculated following Clopper and Pearson [33] with R (https://www.r-project.org/, accessed on 29 June 2021).

### 2.3. Model Analysis

The temporal course for prevalence estimates was calculated using a Bayesian space–time model and BayesX 2.0.1 (http://www.uni-goettingen.de/de/bayesx/550513.html, accessed on 29 June 2021). The logistic regression model allowed to examine the relationship between the probability of disease (prevalence) and potential influencing variables via the logit transformation of the prevalence p (log(p/(1 − p))). Calculations were done for ASFV logit prevalence and logit seroprevalence estimates for hunted wild boar on a monthly basis. Age was categorized in two age classes (<1 years and >1 years). Age was the fixed independent variable and the calculated prevalence estimates constituted the dependent variable. Time, space and season were used as random factors (see the Supplementary Materials, Figures S1 and S2). To incorporate spatial dependencies, the smallest administrative unit of Lithuania, the eldership, was used. The model was adapted from Staubach et al. [34] and is described in more detail in Schulz et al. [28].

### 2.4. Wild Boar Population Data

Wild boar population data were available per hunting season from the 2013/14 hunting season to the 2019/20 hunting season (1 April to 31 March of the following year). These data were estimations based on sightings and snow tracks. In addition, population density estimates based on yearly hunting bag data from 2016 to October 2020 were available. In contrast to the estimations based on sightings and snow tracks, the hunting bag data were available per calendar year (1 January to 31 December of the same year). Both data sets were available at the municipality level as the total numbers of estimated or hunted wild boar, respectively.

For population density calculations, the total number of wild boar per municipality was divided by the area (in km^2^) of the respective municipality. Thus, the estimated population density per municipality is indicated as the number of wild boar per km^2^. Differences in the population density based on estimations and based on the hunting bag number between the different hunting seasons or years, respectively, were calculated using the Kruskal–Wallis test. Assigning potential statistically significant differences to the respective hunting season/year (*p*-value < 0.05), a Mann–Whitney U test was used for pairwise comparisons. Bonferroni correction was applied to control for type I error [35]. To make the population density comparable to the surveillance data and for data mapping, only the population density estimates based on the hunting bag number per year were used in the comparison with the surveillance data.

## 3. Results

### 3.1. Descriptive Analysis of ASF Surveillance Data

**Figure 1:**
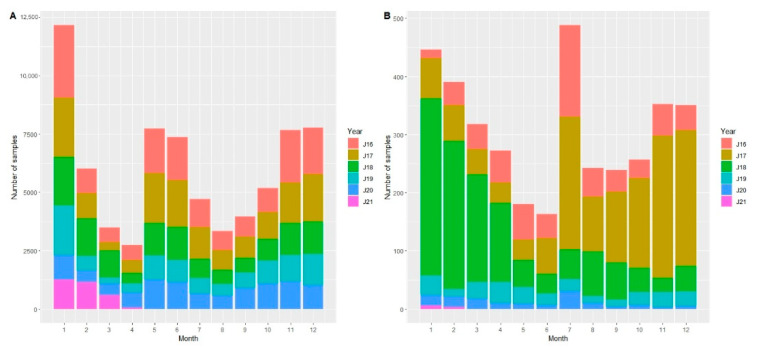
Numbers of samples from hunted wild boar (**A**) and from wild boar found dead (**B**) per month for the years 2016–2021. In 2021, only data from January to 12th April are included.

For the analyses, 75,804 data records were available. Most samples of all the wild boar (hunted and found dead) were investigated in 2017 (18,191 samples), but most samples from hunted animals were retrieved in 2016 (17,284 samples). From 2017 onwards, the total number of samples clearly decreased over time. Particularly, the number of samples obtained from wild boar found dead decreased by more than half from year to year (Figure 1, Table S1). On average, most samples from hunted wild boar were taken in January (*n* = 2101), whereas the lowest average number of hunted animals was sampled in April (*n* = 457). Overall, the proportion of samples from hunted wild boar in the individual months was relatively similar over the years. By contrast, the number of samples originating from wild boar found dead showed the highest values in the months of the second half of 2017, whereas, in 2018, the majority of these samples were taken in the beginning of the year. For wild boar found dead, most samples were taken in July (*n* = 98), whereas the lowest number of samples was obtained in June (*n* = 33) (Figure 1, Table S1).

In all years, the majority of samples originated from wild boar older than one year for both hunted animals and those found dead (Table S1).

**Figure 2:**
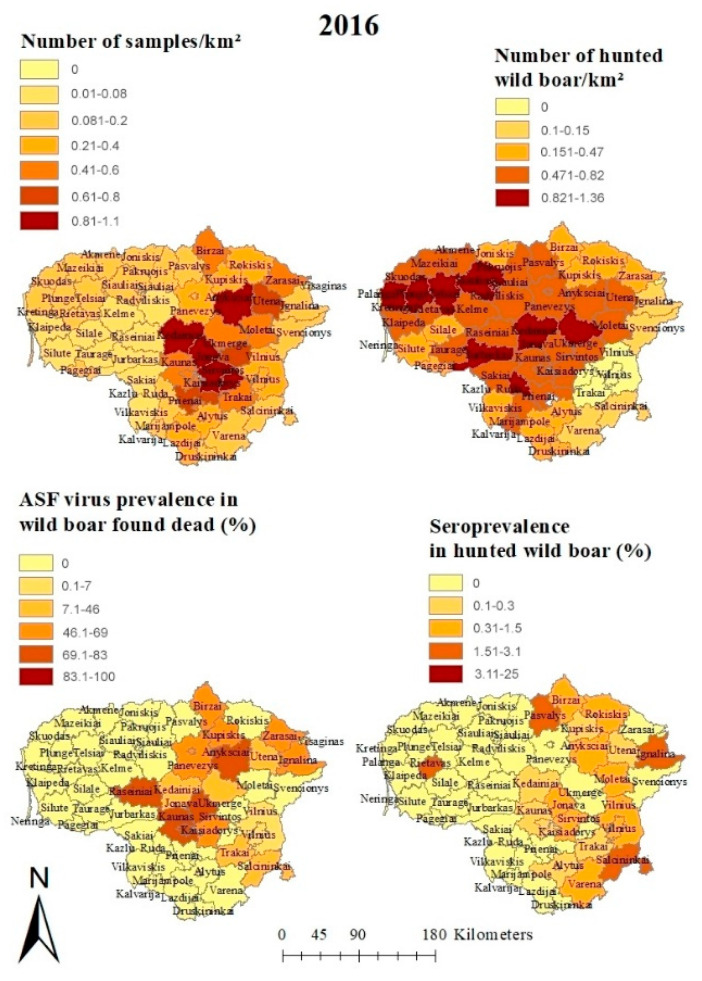
Numbers of investigated ASF samples and hunted wild boar, ASF virus prevalence estimates from wild boar found dead and seroprevalence estimates for hunted wild boar per municipality in Lithuania in 2016.

In 2016 and 2017, most wild boar were sampled in the eastern and central municipalities, particularly in Kedainiai (2016: 1.05 samples/km^2^; 2017: 1.14 samples/km^2^) and Anyksciai (2016: 0.84 samples/km^2^; 2017: 0.63 samples/km^2^). Over the years, the number of samples taken in the east and the center of Lithuania decreased, while those from the western part of the country increased. However, the sample size exceeded 0.75 samples/km^2^ in all of the municipalities after the year 2017 (Figure 2, Figures S3–S6).

### 3.2. Prevalence Estimates

**Figure 3:**
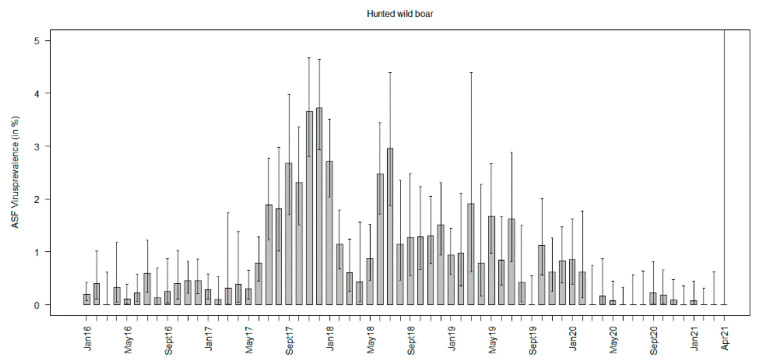
African swine fever virus prevalence estimates per month in hunted wild boar between January 2016 and November 2020.

**Figure 4:**
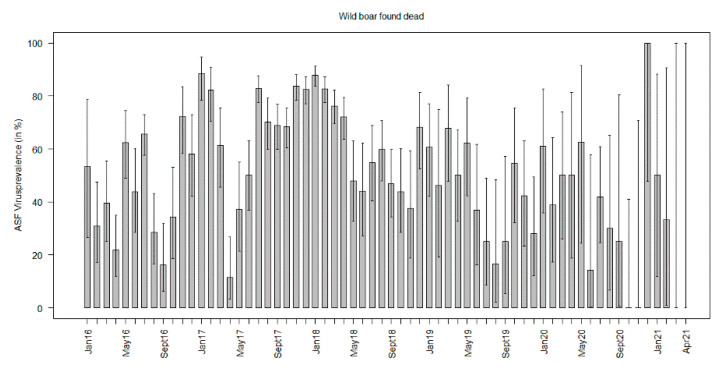
African swine fever virus prevalence estimates per month in wild boar found dead between January 2016 and November 2020.

In almost all months of the study period, ASFV prevalence estimates in wild boar found dead were many times higher than in hunted wild boar. The ASFV prevalence estimates in hunted wild boar increased slightly until the end of 2017 and tended to decrease towards the end of the study period (Figure 3, Table S2). Although no major difference was found in the ASFV prevalence estimates in wild boar found dead over time, higher prevalence estimates with narrow confidence intervals were detected between August 2017 and April 2018. After that, no significant differences in the ASFV prevalence estimates over time were found until the end of the study period (Figure 4, Table S2).

In 2016, only in the municipality Ignalina, located in the eastern part of Lithuania, did the ASFV prevalence estimates in hunted wild boar exceed 1% (4.4%, 95% CI: 1.6–9.4%). In 2017 and 2018, ASFV prevalence estimates in hunted wild boar showed the highest values in municipalities located in the center of the country (Panevezys 2017: 4.1%, 95% CI: 2.8–5.7%, 2018: 14.3%, 95% CI: 7.1–24.7%; Pakruojis 2017: 3.6%, 95% CI: 1.3–7.6%, 2018: 6.8%, 95% CI: 3.7–11.3%). However, in 2018, the highest ASFV prevalence in hunted wild boar was found in Akmene (10.6%, 95% CI: 6.3–16.5%). In 2019 and 2020, ASFV prevalence estimates in hunted wild boar were generally lower. They showed the highest values in the central and western parts of Lithuania (Mazeikiai 2019: 3.1%, 95% CI: 1.6–5.5% 2020: 2.5%, 95% CI: 0.5–7.1%). Until 12 April 2021, only a single ASFV-positive hunted wild boar was found. It was detected in Plunge in western Lithuania (Table S3).

In 2016, ASFV prevalence estimates in wild boar found dead reached values of up to 80%, particularly in the center of Lithuania (Figure 2 and Table S4). As the ASFV prevalence estimates in hunted wild boar, the estimates in wild boar found dead increased in many municipalities, particularly in the center and the west of Lithuania, in 2017 and 2018 (Table S4 and Figure S3). In 2019, only four municipalities showed an ASFV prevalence above 70% in wild boar found dead. In 2020, no dead wild boar were detected in 25 municipalities. In 20 further municipalities, no ASFV-positive wild boar were detected. In Ignalina, a municipality located at the eastern border of the country, a single sample was taken and tested positive (95% CI 2.5–100%). Also, in 2021, the ASFV prevalence was 100% in this municipality with a narrower 95% CI (40–100%). In Mazeikai, located in the west of Lithuania, the ASFV prevalence in wild boar found dead was also high in 2020 (85.7%, 95% CI: 42.1–99.6%). No wild boar were found dead in 2021 in this municipality. In the majority of municipalities, no dead wild boar were detected during the first months of 2021 (Table S4).

**Figure 5:**
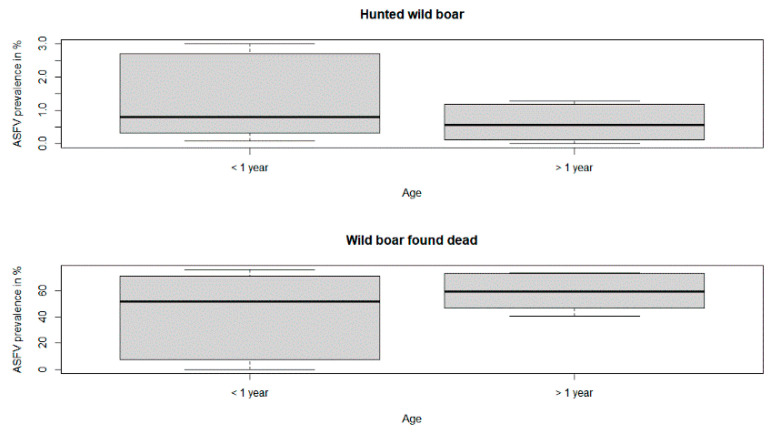
African swine fever virus prevalence estimates of hunted wild boar and wild boar found dead in the two age classes. The horizontal lines that form the top of the boxes illustrate the 75th percentile. The horizontal lines that form the bottom indicate the 25th percentile. The horizontal lines that intersect the box represent the median ASFV prevalence per age class. Whiskers indicate maximum and minimum values that are no more than 1.5 times the span of the interquartile range and the open circles represent outliers, which are single values greater or less than the extremes indicated by the whiskers.

The differences in the yearly ASFV prevalence estimates were not statistically significant in the two age classes, neither in hunted wild boar (*p*-value = 0.42) nor in wild boar found dead (*p*-value = 0.75) (Table 1, Figure 5).

**Figure 6:**
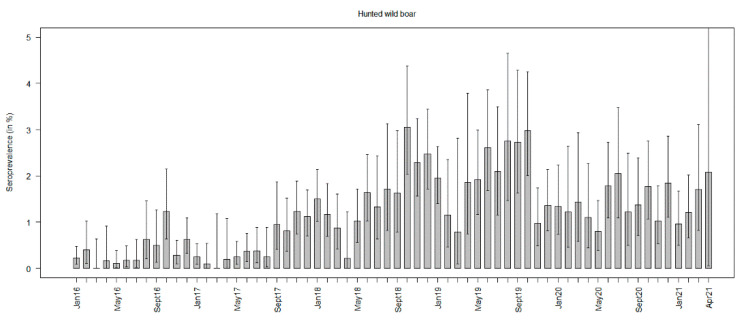
African swine fever virus-specific antibody prevalence estimates (seroprevalence) in hunted wild boar per month from January 2016 to April 2021.

Seroprevalence estimates in hunted wild boar increased over time, showing the highest values in October 2018 (3.1%, 95% CI: 2.0–4.4%) and 2019 (3.0%, 95% CI: 2.0–4.3%). By the end of 2019, they had decreased, but the overlapping confidence intervals suggest that the prevalence estimates in the following study months were not significantly lower than in the two years before (Figure 6, Table S2).

The highest seroprevalence estimate in 2016 was found in Salcininkai (2.6%, 95% CI: 1.0–5.6%), located in the southeast of Lithuania (Table S5 and Figure 2). In 2017, the small easterly municipality of Visaginas in particular showed a high seroprevalence estimate (25%, 95% CI: 0.6–80.6%). However, the 95% confidence intervals were very wide, suggesting a small sample size. In 2017 and 2018, the highest seroprevalence estimates were found in the northern part of central Lithuania (Table S5 and Figures S3 and S4). The number of municipalities with seroprevalence estimates above 1% increased from 24 in 2018 to 31 in 2019. In addition to the centrally located municipalities, some municipalities in the west also showed an increased seroprevalence in hunted wild boar in 2019, 2020 and 2021 (e.g., Mazeikiai 2019: 3.2%, 95% CI: 1.6–5.7%; 2020: 1.7%, 95% CI 0.2–6.0%; 2021: 5.6%, 95% CI: 0.1–27.3%). In 2020, however, the seroprevalence estimates were lower than in the years before in some of the central municipalities (e.g., Birzai 2.1%, 95% CI: 0.6–5.2%) (Table S5 and Figures S5 and S6). In the four analyzed months of 2021, seropositive wild boar were only hunted in 24 municipalities. The highest seroprevalence estimates were detected in municipalities located in the northwest of Lithuania (Siauliai: 9.6%, 95% CI: 4.3–18.1% and Joniskis: 6.7%, 95% CI: 0.2–31.9%) (Table S5).

**Figure 7:**
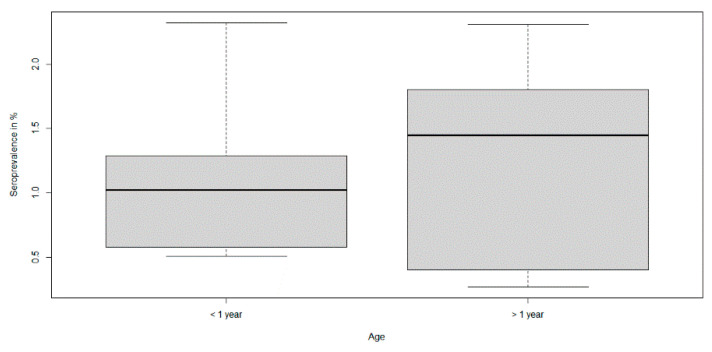
Seroprevalence estimates of hunted wild boar in the two different age classes. The horizontal lines that form the top of the boxes illustrate the 75th percentile. The horizontal lines that form the bottom indicate the 25th percentile. The horizontal lines that intersect the box represent the median seroprevalence per age class. Whiskers indicate maximum and minimum values that are no more than 1.5 times the span of the interquartile range and the open circles represent outliers, which are single values greater or less than the extremes indicated by the whiskers.

The differences in the yearly seroprevalence estimates were not statistically significant when the two age classes were compared (*p*-value = 0.75) (Figure 7). However, the median yearly seroprevalence estimate in animals older than one year showed a slightly higher value than that of the younger animals (Table 1).

### 3.3. Model Analysis

**Figure 8:**
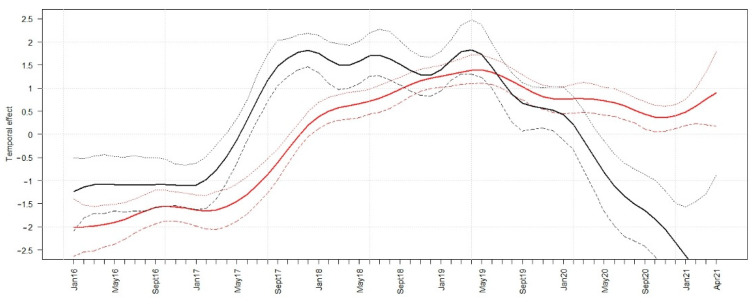
Median temporal effect on the logit prevalence for samples that tested positive for ASFV (black lines) and exclusively seropositive for ASFV-specific antibodies (red lines). The 95% Bayesian credible intervals (BCIs, dashed lines) are indicated for ASFV-positive wild boar (black) and animals exclusively seropositive for ASFV-specific antibodies (red).

The model analyses showed an increase in the temporal trend of the logit prevalence of ASFV-positive hunted wild boar from February 2017 until October 2018. In May 2019 and in the subsequent study months, the logit prevalence decreased (Figure 8). The temporal trend of the logit seroprevalence of hunted wild boar started to increase a few months later than the logit ASFV prevalence and the rise was less pronounced. Starting in May 2019, the logit seroprevalence seemed to decrease slightly, but rose again by the end of the study period (Figure 8).

### 3.4. Wild Boar Population Data

**Figure 9:**
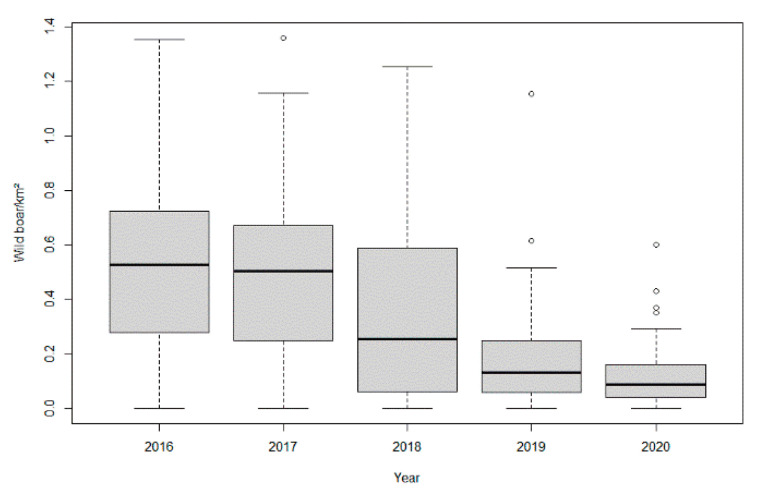
Estimated wild boar population density (wild boar/km^2^) based on hunting bag data per year. The horizontal lines that form the top of the boxes illustrate the 75th percentile. The horizontal line that forms the bottom indicate the 25th percentile. The horizontal lines that intersect the box represent the median number of wild boar per square kilometer. Whiskers indicate maximum and minimum values that are no more than 1.5 times the span of the interquartile range and the open circles represent outliers, which are single values greater or less than the extremes indicated by the whiskers.

The estimated wild boar population density based on sightings and snow tracks decreased over time. The estimated population density in the 2018/19 hunting season was statistically significantly lower than in the previous hunting seasons. However, in the 2019/2020 hunting season, the population density was only significantly lower than the one estimated for 2013/14 and 2014/15 (Table S6 and Figure S7). Estimated population density data based on hunting bags were only available after 2016 and on a yearly base. The number of wild boar/km^2^ calculated on the basis of the hunting bags decreased significantly over time. In 2020, it was significantly lower than in 2016, 2017 and 2018 (Table S7 and Figure 9).

In all years, the estimated population density based on hunting bag data showed the highest values mainly in the western municipalities of Lithuania. However, in 2016 and 2017, a few municipalities in the center also had a population density above 0.8 wild boar/km^2^. In 2019, the population density was higher than 1 wild boar/km (1.15) only in Kretinga, a municipality in the west of Lithuania with a commercial hunting ground that is considered free from ASF. The highest population density value was also reported from this municipality in 2020, although it was somewhat lower than in the year before (0.60 wild boar/km^2^) (Figure 2 and Figures S3–S6).

## 4. Discussion

Several studies have described the epidemiological situation of ASF in Lithuania in certain periods [9,10,25]. In the present study, all available and evaluable ASF wild boar surveillance data were used to illustrate the course of the disease over time and to obtain an overview of the current epidemiological ASF situation in wild boar in Lithuania. In addition, surveillance efforts were described and the wild boar population density estimated for each year individually. Therefore, a comprehensive description of the epidemiological situation concerning ASF in wild boar in Lithuania was possible and several influencing factors could be taken into consideration.

The huge numbers of samples that were available for the analysis show the strong effort of Lithuanian authorities to combat the disease. Mačiulskis, et al. [9] showed an increase of the number of investigated samples over time starting in 2014, with a peak in 2016 and 2017. This peak was discussed as a result of paying incentives to hunters and other people who reported finding dead wild boar since 2016. This study confirms these results. The large number of samples originating from wild boar found dead in 2017 might have been due to an increased awareness, incentives for reporting and disposal and the willingness of hunters and the public to support passive surveillance. Moreover, the estimated wild boar population was still high at that time. Thus, the large number of susceptible animals combined with the high case/fatality ratio of ASF could have led to a large number of detected dead wild boar. The huge number of wild boar found dead indicates a high viral load in the environment and could thus explain the high number of ASF outbreaks in domestic pig holdings in 2018. The subsequent constant decrease in the numbers of samples during the following years was certainly due to the decrease of the susceptible wild boar population, but signs of fatigue within the hunters’ community may have played a role, too. In a recent study, however, it was found that Lithuanian hunters claimed to be willing to support ASF control measures with increased hunting, indicating rather that the lower number of wild boar was the reason for the lower sample size. In contrast, passive surveillance (i.e., the detecting, reporting and sometimes also the disposal of detected wild boar carcasses by burial) was not very popular among the participating hunters [36], supporting the assumption that the decreasing number of samples from wild boar found dead could be due to the lack of hunters’ motivation. Similar decreasing trends in sample sizes over time were also found in the other two Baltic states, Estonia and Latvia [24,27]. In 2021, data were only available until the 12th April, but in the first three months of 2021, the number of samples from hunted animals seemed to increase again compared to the numbers in the same months of the previous year. This may indicate a slow recovery of the wild boar population. The population density data also revealed a stabilization of the number of wild boar/km^2^ in 2020 compared to 2019.

The usually larger number of samples of hunted wild boar in January and the lower numbers in April are not surprising. January is part of the main hunting season, involving driven hunts, while in April hunting activities are usually suspended due to ethical reasons (it is the main reproduction season of wild boar).

On average, most dead wild boar were detected in July, which may have been due to increased leisure activities in the forests during summer, thus resulting in a higher detection probability. Moreover, most of wild boar hide in crop fields at this time of the year, which may lead to an increased probability of direct and indirect contacts between different groups of wild boar and result in an increased probability of disease spread. The peak of detected dead wild boar in July may have been due to the fact that there are more susceptible wild boar in summer due to the main reproduction period occurring in spring and an increased case/fatality ratio of ASF in young wild boar. However, the low average number of wild boar found dead in June contradicts these explanations, as it can be assumed that the external circumstances do not differ to a great extent between June and July. Probst et al. [37] found that wild boar carcasses decompose much faster in the warm season and that mammalian scavengers visit the carcasses more frequently in summer. Dead wild boar may therefore disappear faster from the environment in this season, which might explain the low numbers of dead wild boar detected in June. In Poland, similar patterns were found and the numbers of samples from passive surveillance were high in July, while it seems that they were significantly lower in June [38].

Most samples came from animals that were estimated to be older than one year. This is not surprising, since animals between one and two years usually constitute the main target group for hunters [16,39,40]. Similar results were also obtained in other countries [27,41]. The spatial shift in the numbers of samples collected towards the west suggests that sampling was increasingly performed in areas where ASF was mainly present in the respective year. The population density decreased over time, particularly in the areas affected in the beginning of the epidemic, which could be an additional explanation for the decrease in the number of samples taken in these municipalities.

ASFV prevalence estimates were separately performed for hunted wild boar and wild boar found dead. The ASFV prevalence estimates in hunted wild boar reflect the true prevalence within the wild boar population much more accurately than the ASFV prevalence estimates in wild boar found dead. The probability that a wild boar found dead in an ASF-endemic region will be ASF-positive is much higher than for an apparently healthy wild boar that has been shot. This may lead to an overestimate of the true ASFV prevalence among wild boar found dead. Detected wild boar carcasses are often decomposed, which may impair the quality of samples and ultimately lead to an underestimation of the true prevalence. In essence, the uncertainty of the estimate is substantially increased for samples from wild boar found dead. All wild boar and wild boar carcasses are regularly tested for the presence of the ASFV genome, but samples obtained from (heavily decomposed) carcasses are not usually tested for ASFV-specific antibodies. Thus, to ensure consistency, seroprevalence estimates were only calculated for hunted wild boar.

The much higher ASFV prevalence estimates in wild boar found dead are not surprising, since several studies have demonstrated the high case/fatality ratio of ASF and the resulting higher probability of obtaining ASFV-positive samples from wild boar found dead, as already mentioned [20,21,22,26,42]. The findings of the present study also confirm the results of previous analyses of Lithuanian surveillance data [9]. Only data from 2016 and later were used for the analyses, since data records from previous years were often incomplete and had to be excluded. However, the epidemic had already started in Lithuania in 2014; thus, ASF had been spreading for two years in 2016 when we started this analysis. Pautienius et al. [10] and Mačiulskis et al. [9] already observed an increase in the ASFV prevalence from the beginning of the epidemic until 2017 and 2018. This temporal trend could be confirmed by our analyses, but it seems that the ASFV prevalence in hunted and wild boar found dead declined in recent years. This course of the disease may be explained by the decreasing number of susceptible wild boar and the resulting slowdown of disease spread. However, the wide confidence intervals, particularly in wild boar found dead, indicate a small sample size and may explain why the results lack statistical significance.

The findings concerning the course of the disease in wild boar were also confirmed by our model analysis. We included the prevalence estimates for neighboring administrative units and time points in a Bayesian model. Thus, the modeling results may have yielded more robust estimates, which are less prone to bias. We used the smallest administrative unit, i.e., the eldership, as the reference in our spatial analysis and calculated only the temporal course of the logit prevalence for ASFV- and seroprevalence for hunted animals. The logit transformation of the prevalence was the best way to demonstrate the variation of the prevalence. The number of samples originating from wild boar found dead per eldership was too small to yield reliable model results.

The increasing seroprevalence in hunted animals, which showed a slight delay when compared to the ASFV genome prevalence, and the subsequent slight decrease indicate that ASF took a similar course in Lithuania as observed in other countries [24,27,28]. However, in the last few months of the study period, the seroprevalence seemed to increase again. Moreover, the detection of ASFV in hunted animals even in late 2020 and early 2021 proves that the virus is still circulating, although the prevalence may be very low, resulting in a clearly decreased detection probability. The risks that attenuated virus strains may circulate or that surviving animals might shed the infectious virus (“carriers”) are strongly disputed and cannot be excluded as hypothetical explanations for the observed epidemiological course of ASF in Lithuania [29,30,31,43,44]. It should be noted, however, that the existence of ASF “carriers” has been controversially discussed [29,30].

In contrast to previous findings, the present analyses did not show any significant differences in the prevalence estimations for the two age classes [20,21,24], suggesting that surveillance efforts should include all age classes equally according to the current knowledge. However, there is still an urgent need to continue data analyses, in other ASF-affected countries also, in order to evaluate the age distribution in various prevalence estimates further and thus to support the design of targeted surveillance measures or allow for their adaptation in an evidence-based manner.

Wild boar population density estimates are always accompanied by limitations. Also, hunting bag data does not represent the true population [45,46]. In Lithuania, every hunting club can determine the targeted number of wild boar to be hunted. There are no governmental regulations regarding the numbers of wild boar that should be hunted during the hunting season. Since the start of the epidemic in 2014, hunting rules were amended in Lithuania and wild boar could be hunted all year, irrespective of the age or sex of the animals. In our study, we compared numbers based on the same methods over the years to obtain estimates that are as reliable as possible. When calculating the numbers of wild boar in the individual municipalities, we assumed an even distribution of the population within the municipality and did not include landscape features, which may affect the distribution.

Similar to previous studies, the wild boar density decreased over time [20,24,41]. It can be assumed that this reduction of the number of wild boar was mainly due to the high case/fatality ratio of ASF [39,47]. In all analyzed years, the wild boar density was higher in the west of Lithuania, which is probably due to the start of the epidemic in the eastern part of the country and to the intensive hunting of wild boar, including females, in that part of the country. In 2016 and 2017 particularly, the numbers of samples per municipality diverged from the number of hunted wild boar per municipality and were higher in central Lithuania. This might have been due to increased surveillance efforts in ASF-affected areas and the accompanying high ASFV prevalence estimates. Over time, a shift towards the west could be observed and, in the last few years, only seroprevalence estimates showed higher values in the center of Lithuania. A similar epidemiological course of the disease was observed in Estonia and Latvia, where ASF was also introduced into the eastern part of the respective countries, presumably from Belarus or the Russian Federation, respectively, and spread towards the west over time [20,21,28].

Despite the comparable epidemiology of ASF in wild boar and its temporal and spatial course, an elimination of the disease in Lithuania is currently not within reach. Possibly, the occurrence of ASF outbreaks in Lithuanian domestic pig holdings—which mainly take place in the newly affected areas or in the areas with continuous circulation of ASFV in the wild boar population—and thus the potential indirect transmission between wild boar and domestic pigs and vice versa, might play a role in this difficult situation. Furthermore, it is still common in rural areas of Lithuania to keep a small number of pigs in backyards at a level of biosafety/biosecurity that is not sufficient to prevent the introduction of ASFV. It is obvious that pigs in these holdings are prone to become infected with ASF, thus feeding the epidemic [15]. However, in Estonia, where all backyard farms were eliminated, no outbreaks in domestic pig farms have been reported since 2017 and no ASFV-positive wild boar were detected for more than one year, new ASFV-positive wild boar cases were reported in August 2020 [24,48]. Thus, engagement, motivation and the willingness of all involved stakeholders must be continuously maintained to keep all surveillance and control efforts at a high level and the disease situation needs to be permanently monitored and analyzed to obtain the knowledge required to adapt surveillance and control as needed. Moreover, open questions like the potential role of surviving animals and of attenuated viruses, the threat of re-introduction from neighboring countries, the possibilities of disease detection at a very low prevalence and the effectiveness of different control measures need to be addressed.

## Figures and Tables

**Table 1:** Median yearly African swine fever virus and seroprevalence estimates in Lithuanian wild boar from 2016–April 2021 for each age class.

	Median ASFV Prevalence in Hunted Wild Boar in %	Median ASFV Prevalence in Wild Boar Found Dead in %	Median Seroprevalence in Hunted Wild Boar in %
<1 year	0.80	51.75	1.02
>1 year	0.56	59.23	1.45

## Data Availability

The original data used for the analyses can be obtained from the authors after approval by the responsible institution in Lithuania.

## Supplementary Materials

The following are available online at https://www.mdpi.com/article/10.3390/v13071276/s1, Figure S1: Median seasonal effect on the logit prevalence of samples obtained from hunted wild boar in Lithuania that tested PCR-positive, irrespective of the serological result. 95% Bayesian credible intervals (BCIs) are indicated (dotted lines). Figure S2: Median seasonal effect of samples that had tested exclusively serologically positive on the logit prevalence. 95% Bayesian credible intervals (BCIs) are indicated (dotted lines). Figure S3: Numbers of investigated ASF samples, numbers of hunted wild boar, ASF virus prevalence estimates from wild boar found dead and seroprevalence estimates for hunted wild boar per municipality in Lithuania in 2017. Figure S4: Numbers of investigated ASF samples, numbers of hunted wild boar, ASF virus prevalence estimates for wild boar found dead and seroprevalence estimates for hunted wild boar per municipality in Lithuania in 2018. Figure S5: Numbers of investigated ASF samples, numbers of hunted wild boar, ASF virus prevalence estimates for wild boar found dead and seroprevalence estimates for hunted wild boar per municipality in Lithuania in 2019. Figure S6: Numbers of investigated ASF samples, numbers of hunted wild boar, ASF virus prevalence estimates for wild boar found dead and seroprevalence estimates for hunted wild boar per municipality in Lithuania in 2020. Figure S7: Estimated wild boar population density (wild boar/km^2^) based on sightings and snow tracks per hunting season. The horizontal lines that form the top of the boxes illustrate the 75th percentile. The horizontal lines that form the bottom indicate the 25th percentile. The horizontal lines that intersect the box represent the median number of wild boar per square kilometer. Whiskers indicate maximum and minimum values that are no more than 1.5 times the span of the interquartile range and the open circles represent outliers, which are single values greater or less than the extremes indicated by the whiskers. Table S1: Numbers of samples originating from wild boar hunted or found dead in Lithuania per age class, year and month. Table S2: ASF virus prevalence and seroprevalence estimates for hunted wild boar and ASF virus prevalence estimates for wild boar found dead, including the 95% confidence intervals, on a monthly basis from January 2016 until April 2021. Blank fields indicate a lack of data in the respective month. Table S3: ASF virus prevalence of hunted wild boar, including the 95% confidence intervals, at the municipality level in the years 2016–2021 (only the first 4 months for 2021). Blank fields indicate a lack of data for the respective municipality. Table S4: ASF virus prevalence for wild boar found dead, including the 95% confidence intervals, at the municipality level in the years 2016–2021 (only the first 4 months for 2021). Blank fields indicate a lack of data for the respective municipality. Table S5: Seroprevalence in hunted wild boar, including the 95% confidence intervals, at the municipality level in the years 2016–2021 (only the first 4 months for 2021). Blank fields indicate a lack of data for the respective municipality. Table S6: Median of estimated number of wild boar/km^2^ based on sightings and snow tracks per hunting year and the results of the Mann–Whitney U test. Significant differences between the hunting seasons are highlighted. Table S7: Median of the estimated number of wild boar/km^2^ based on hunting bag data per year and the results of the Mann–Whitney U test. Significant differences between the hunting seasons are highlighted.
