# Supplementary material for: African Swine Fever and Its Epidemiological Course in Lithuanian Wild Boar
Source: Viruses. 2021 Jun 30;13(7):1276. doi: 10.3390/v13071276 (PMC8310040; doi:10.3390/v13071276)
Supplement: Supplementary file 1 [file viruses-13-01276-s001.zip › viruses-1255391-supplementary-final.pdf]

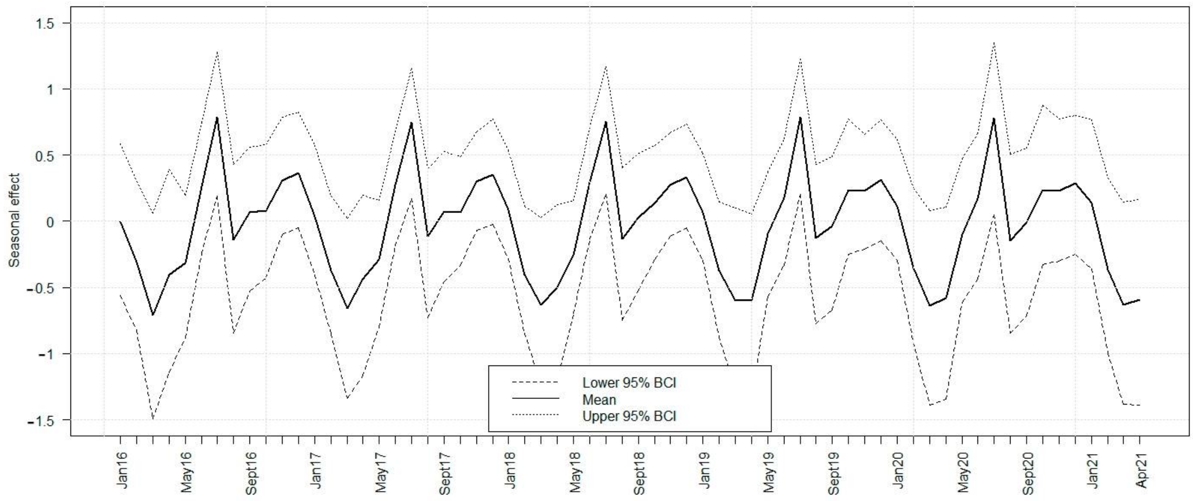

Figure S1. Median seasonal effect on the logit prevalence of samples obtained from hunted wild boar in Lithuania that tested PCR-positive, irrespective of the serological result. 95% Bayesian credible intervals (BCI) are indicated (dotted lines).

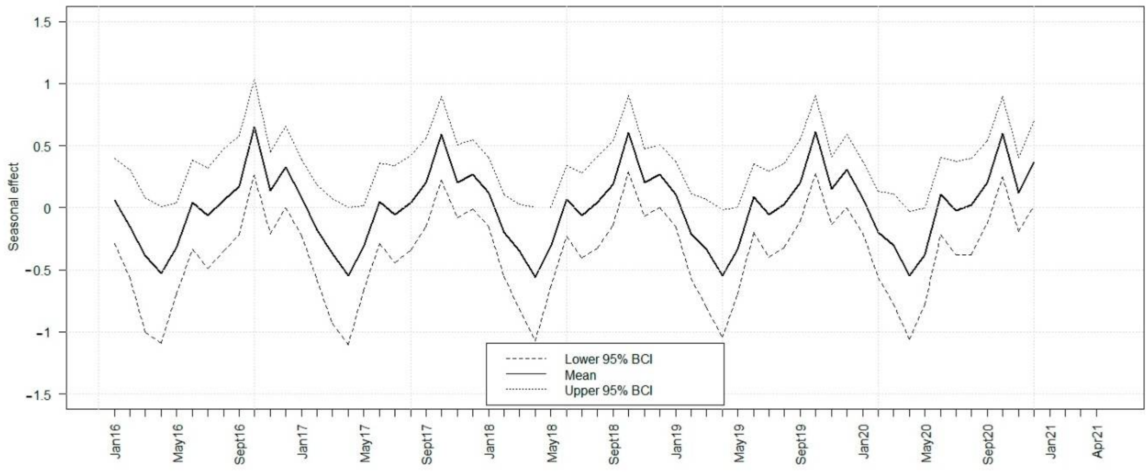

Figure S2. Median seasonal effect of samples that had tested exclusively serologically positive on the logit prevalence. 95% Bayesian credible intervals (BCI) are indicated (dotted lines).

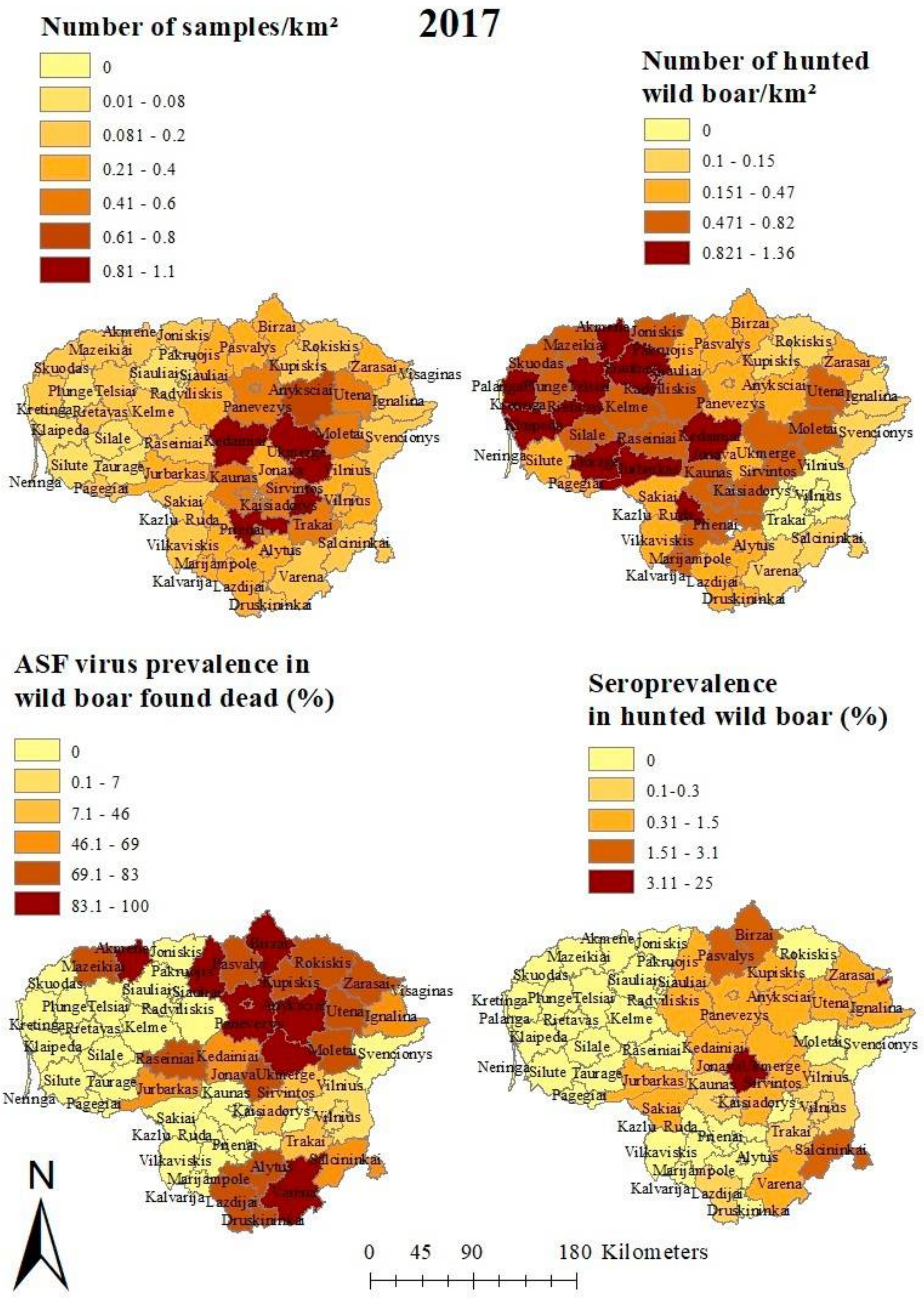

Figure S3. Numbers of investigated ASF samples, numbers of hunted wild boar, ASF virus prevalence estimates for wild boar found dead and seroprevalence estimates for hunted wild boar per municipality in Lithuania and in 2017.

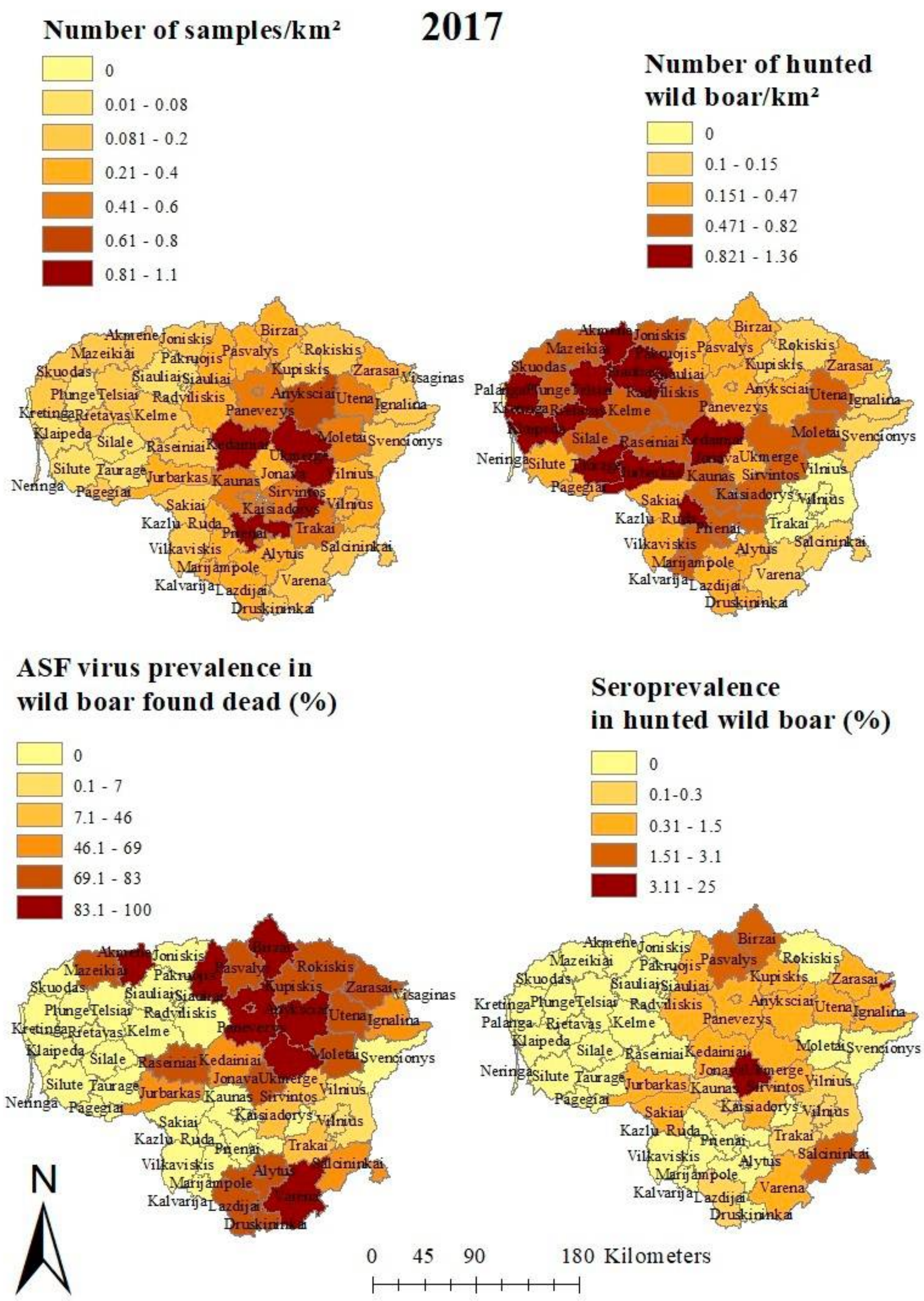

Figure S4. Numbers of investigated ASF samples, numbers of hunted wild boar, ASF virus prevalence estimates for wild boar found dead and seroprevalence estimates for hunted wild boar per municipality in Lithuania and in 2018.

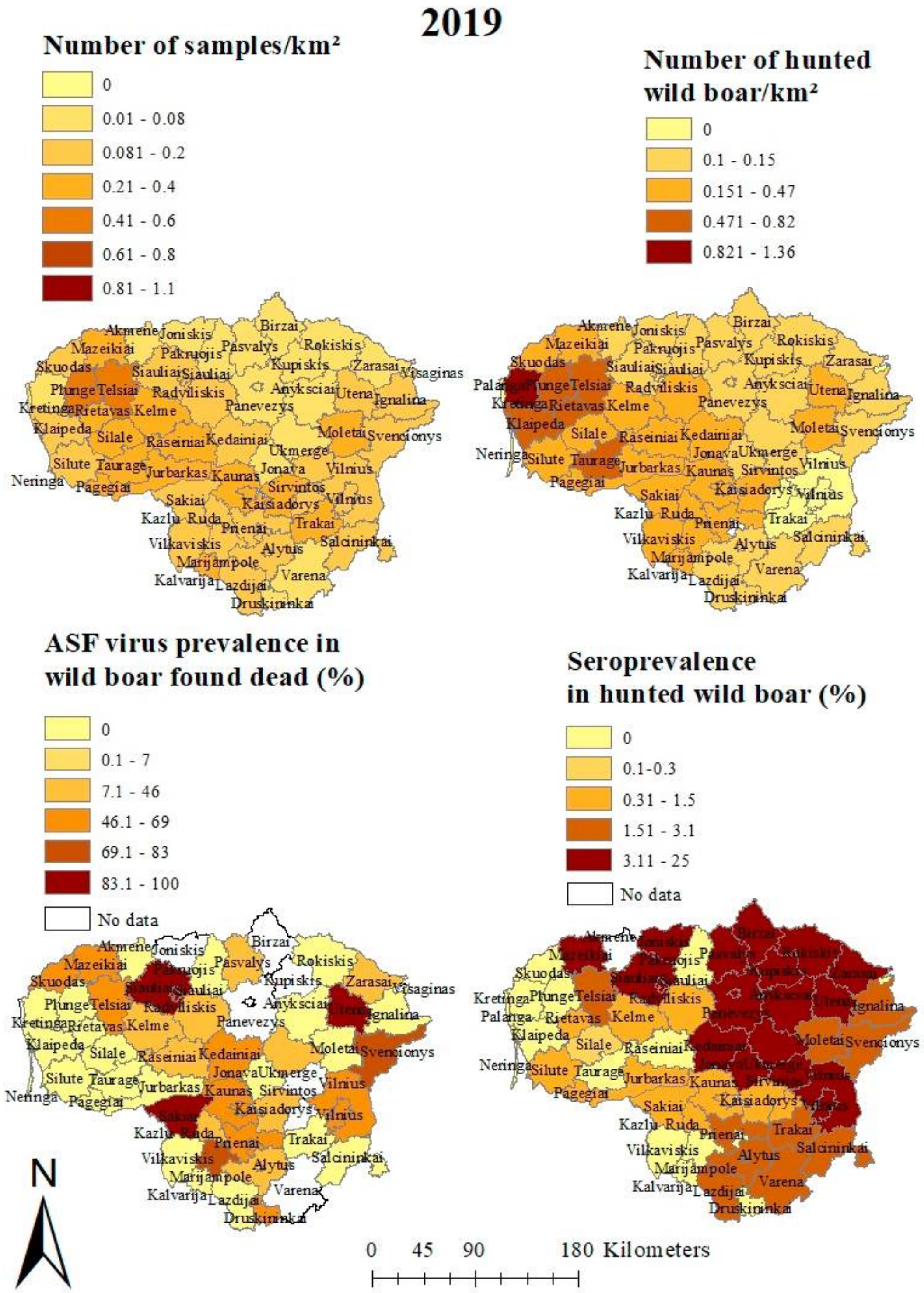

Figure S5. Numbers of investigated ASF samples, numbers of hunted wild boar, ASF virus prevalence estimates for wild boar found dead and seroprevalence estimates for hunted wild boar per municipality in Lithuania and in 2019.

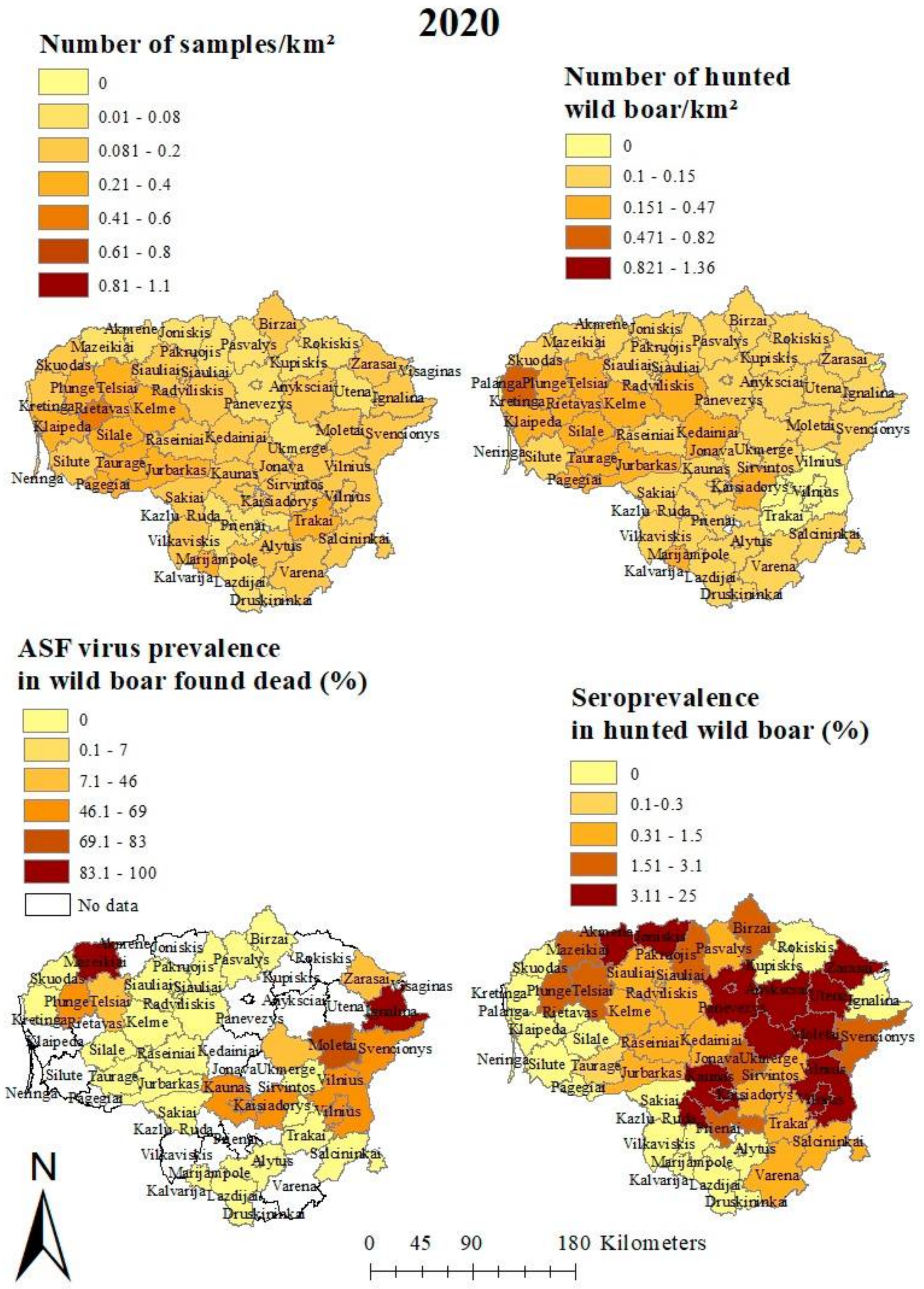

Figure S6. Numbers of investigated ASF samples, numbers of hunted wild boar, ASF virus prevalence estimates for wild boar found dead and seroprevalence estimates for hunted wild boar per municipality in Lithuania and in 2020.

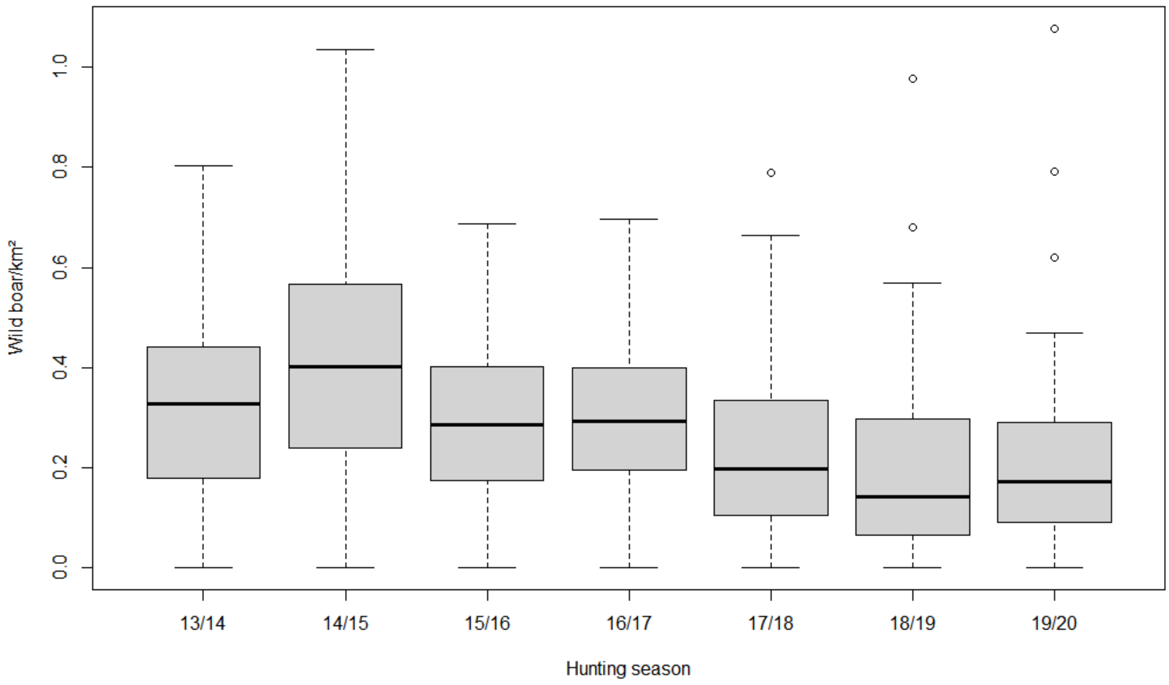

Figure S7. Estimated wild boar population density (wild boar/km^2) based on sightings and snow tracks per hunting season. The horizontal lines that form the top of the boxes illustrate the 75th percentile. The horizontal lines that form the bottom indicate the 25th percentile. The horizontal lines that intersect the box represent the median number of wild boar per square kilometer. Whiskers indicate maximum and minimum values that are no more than 1.5 times the span of the interquartile range and the open circles represent outliers, which are single values greater or less than the extremes indicated by the whiskers.

Table S1. Numbers of samples originating from wild boar hunted or found dead in Lithuania per age class, year and month.

	Year	2016			2017			2018			2019			2020			2021			Average total
**Month**	Age class / Origin	<1	>1	**Total**	<1	>1	**Total**	<1	>1	**Total**	<1	>1	**Total**	<1	>1	**Total**	<1	>1	**Total**	
**1**	**Total**	1195	1922	**3117**	1147	1482	**2629**	543	1827	**2370**	751	1412	**2163**	572	500	**1072**	620	636	**1256**	**2101**
	Hunted	1189	1913	**3102**	1125	1435	**2560**	456	1609	**2065**	746	1384	**2130**	569	485	**1054**	618	632	**1250**	**2027**
	Found dead	6	9	**15**	22	47	**69**	87	218	**305**	5	28	**33**	3	15	**18**	2	4	**6**	**74**
**2**	**Total**	373	711	**1084**	474	697	**1171**	461	1394	**1855**	182	448	**630**	240	269	**509**	530	631	**1161**	**1068**
	Hunted	355	690	**1045**	456	653	**1109**	398	1202	**1600**	180	437	**617**	233	258	**491**	529	629	**1158**	**1003**
	Found dead	18	21	**39**	18	44	**62**	63	192	**255**	2	11	**13**	7	11	**18**	1	2	**3**	**65**
**3**	Total	123	541	**664**	123	275	**398**	281	1066	**1347**	68	222	**290**	238	271	**509**	217	371	**588**	**633**
	Hunted	104	517	**621**	116	238	**354**	228	934	**1162**	62	200	**262**	236	255	**491**	217	371	**588**	**580**
	Found dead	19	24	**43**	7	37	**44**	53	132	**185**	6	22	**28**	2	16	**18**				**64**
**4**	Total	59	642	**701**	64	523	**587**	42	557	**599**	66	353	**419**	190	457	**647**	18	42	**60**	**502**
	Hunted	50	596	**646**	57	495	**552**	20	443	**463**	63	320	**383**	188	449	**637**	18	42	**60**	**457**
	Found dead	9	46	**55**	7	28	**35**	22	114	**136**	3	33	**36**	2	8	**10**				**54**
**5**	Total	133	1838	**1971**	130	2039	**2169**	116	1323	**1439**	101	953	**1054**	138	1137	**1275**				**1582**
	Hunted	103	1807	**1910**	121	2013	**2134**	108	1285	**1393**	100	925	**1025**	138	1128	**1266**				**1546**
	Found dead	30	31	**61**	9	26	**35**	8	38	**46**	1	28	**29**		9	**9**				**36**
**6**	Total	59	1807	**1866**	116	1975	**2091**	46	1406	**1452**	63	900	**963**	73	1076	**1149**				**1504**
	Hunted	46	1779	**1825**	87	1942	**2029**	40	1378	**1418**	58	886	**944**	73	1069	**1142**				**1472**
	Found dead	13	28	**41**	29	33	**62**	6	28	**34**	5	14	**19**		7	**7**				**33**
**7**	Total	167	1184	**1351**	227	1385	**1612**	52	796	**848**	49	651	**700**	52	630	**682**				**1039**
	Hunted	75	1119	**1194**	111	1272	**1383**	42	755	**797**	46	634	**680**	47	604	**651**				**941**
	Found dead	92	65	**157**	116	113	**229**	10	41	**51**	3	17	**20**	5	26	**31**				**98**

**8**	Total	145	715	**860**	167	791	**958**	123	563	**686**	79	412	**491**	97	494	**591**				**717**
	Hunted	114	697	**811**	117	747	**864**	92	517	**609**	75	404	**479**	96	485	**581**				**669**
	Found dead	31	18	**49**	50	44	**94**	31	46	**77**	4	8	**12**	1	9	**10**				**48**
**9**	Total	238	660	**898**	274	759	**1033**	191	503	**694**	183	491	**674**	289	606	**895**				**839**
	Hunted	222	639	**861**	209	702	**911**	168	462	**630**	177	485	**662**	288	604	**892**				**791**
	Found dead	16	21	**37**	65	57	**122**	23	41	**64**	6	6	**12**	1	3	**3**				**48**
**10**	Total	400	648	**1048**	391	915	**1306**	344	632	**976**	406	594	**1000**	432	665	**1097**				**1085**
	Hunted	386	630	**1016**	311	840	**1151**	330	605	**935**	396	582	**978**	429	661	**1090**				**1034**
	Found dead	14	18	**32**	80	75	**155**	14	27	**41**	10	12	**22**	3	4	**7**				**51**
**11**	Total	976	1329	**2305**	617	1353	**1970**	515	889	**1404**	529	632	**1161**	579	599	**1178**				**1604**
	Hunted	948	1303	**2251**	506	1219	**1725**	509	871	**1380**	521	614	**1135**	576	599	**1175**				**1533**
	Found dead	28	26	**54**	111	134	**245**	6	18	**24**	8	18	**26**	3		**3**				**70**
**12**	Total	920	1125	**2045**	668	1599	**2267**	554	875	**1429**	626	728	**1354**	569	467	**1036**				**1626**
	Hunted	887	1115	**2002**	536	1497	**2033**	545	840	**1385**	615	714	**1329**	569	462	**1031**				**1556**
	Found dead	33	10	**43**	132	102	**234**	9	35	**44**	11	14	**25**		5	**5**				**70**
	Total	4788	13122	**17910**	4398	13793	**18191**	3268	11831	**15099**	3103	7796	**10899**	3469	7171	**10640**	1385	1680	**3065**	

Table S2. ASF virus prevalence and seroprevalence estimates for hunted wild boar and ASF virus prevalence estimates for wild boar found dead including the 95% confidence intervals on a monthly basis from January 2016 until April 2021. Blank fields indicate lack of data in the respective month.

	ASFV prevalence in hunted wild boar			ASFV prevalence in wild boar found dead			Seroprevalence in hunted wild boar		
Study month	**Prevalence in %**	**Lower 95% CI in %**	**Upper 95% CI in %**	**Prevalence in %**	**Lower 95% CI in %**	**Upper 95% CI in %**	**Prevalence in %**	**Lower 95% CI in %**	**Upper 95% CI in %**
Jan 16	0.2	0.1	0.4	53.3	26.6	78.7	0.2	0.1	0.5
Feb 16	0.4	0.1	1.0	30.8	17.0	47.6	0.4	0.1	1.0
Mar16	0.0	0.0	0.6	39.5	25.0	55.6	0.0	0.0	0.6
Apr 16	0.3	0.0	1.2	21.8	11.8	35.0	0.2	0.0	0.9
May 16	0.1	0.0	0.4	62.3	49.0	74.4	0.1	0.0	0.4
Jun 16	0.2	0.1	0.6	43.9	28.5	60.3	0.2	0.0	0.5
Jul 16	0.6	0.2	1.2	65.6	57.6	73.0	0.2	0.0	0.6
Aug 16	0.1	0.0	0.7	28.6	16.6	43.3	0.6	0.2	1.5
Sep 16	0.2	0.0	0.9	16.2	6.2	32.0	0.5	0.1	1.3
Oct 16	0.4	0.1	1.0	34.4	18.6	53.2	1.2	0.6	2.1
Nov 16	0.4	0.2	0.8	72.2	58.4	83.5	0.3	0.1	0.6
Dec 16	0.5	0.2	0.9	58.1	42.1	73.0	0.6	0.3	1.1
Jan 17	0.3	0.1	0.6	88.4	78.4	94.9	0.2	0.1	0.5
Feb 17	0.1	0.0	0.5	82.3	70.5	90.8	0.1	0.0	0.5
Mar 17	0.3	0.0	1.7	61.4	45.5	75.6	0.0	0.0	1.2
Apr 17	0.4	0.0	1.4	11.4	3.2	26.7	0.2	0.0	1.1
May 17	0.3	0.1	0.6	37.1	21.5	55.1	0.3	0.1	0.6
Jun 17	0.8	0.4	1.3	50.0	37.0	63.0	0.4	0.1	0.8
Jul 17	1.9	1.2	2.8	83.0	77.5	87.6	0.4	0.1	0.9
Aug 17	1.8	1.0	3.0	70.2	59.9	79.2	0.2	0.0	0.9
Sep 17	2.7	1.7	4.0	68.9	59.8	76.9	1.0	0.4	1.9
Oct 17	2.3	1.5	3.4	68.4	60.4	75.6	0.8	0.4	1.5
Nov 17	3.7	2.8	4.7	83.7	78.4	88.1	1.2	0.8	1.9
Dec 17	3.7	2.9	4.6	82.5	77.0	87.1	1.1	0.7	1.7
Jan 18	2.7	2.0	3.5	87.9	83.7	91.3	1.5	1.0	2.1
Feb 18	1.1	0.7	1.8	82.7	77.5	87.2	1.2	0.7	1.8
Mar 18	0.6	0.2	1.2	76.2	69.4	82.2	0.9	0.4	1.6
Apr 18	0.4	0.1	1.6	72.1	63.7	79.4	0.2	0.0	1.2
May 18	0.9	0.5	1.5	47.8	32.9	63.1	1.0	0.6	1.7
Jun 18	2.5	1.7	3.4	44.1	27.2	62.1	1.6	1.0	2.5
Jul 18	3.0	1.9	4.4	54.9	40.3	68.9	1.3	0.6	2.4
Aug 18	1.1	0.5	2.4	59.7	47.9	70.8	1.7	0.8	3.1
Sep 18	1.3	0.5	2.5	46.9	34.3	59.8	1.6	0.8	3.0
Oct 18	1.3	0.7	2.2	43.9	28.5	60.3	3.1	2.0	4.4
Nov 18	1.3	0.8	2.1	37.5	18.8	59.4	2.3	1.6	3.2
Dec18	1.5	0.9	2.3	68.2	52.4	81.4	2.5	1.7	3.4
Jan 19	0.9	0.6	1.4	60.6	42.1	77.1	2.0	1.4	2.6
Feb 19	1.0	0.4	2.1	46.2	19.2	74.9	1.2	0.5	2.4
Mar 19	1.9	0.6	4.4	67.9	47.6	84.1	0.8	0.1	2.8
Apr 19	0.8	0.2	2.3	50.0	32.9	67.1	1.9	0.7	3.8
May 19	1.7	1.0	2.7	62.1	42.3	79.3	1.9	1.2	3.0
Jun 19	0.8	0.4	1.7	36.8	16.3	61.6	2.6	1.7	3.9
Jul 19	1.6	0.8	2.9	25.0	8.7	49.1	2.1	1.2	3.5
Aug 19	0.4	0.1	1.5	16.7	2.1	48.4	2.7	1.5	4.7
Sep 19	0.0	0.0	0.6	25.0	5.5	57.2	2.7	1.6	4.3
Oct 19	1.1	0.6	2.0	54.5	32.2	75.6	3.0	2.0	4.3

Nov 19	0.6	0.2	1.3	42.3	23.4	63.1	1.0	0.5	1.7
Dec 19	0.8	0.4	1.5	28.0	12.1	49.4	1.4	0.8	2.1
Jan 20	0.9	0.4	1.6	61.1	35.7	82.7	1.3	0.7	2.2
Feb 20	0.6	0.1	1.8	38.9	17.3	64.3	1.2	0.5	2.6
Mar 20	0.0	0.0	0.7	50.0	26.0	74.0	1.4	0.6	2.9
Apr 20	0.2	0.0	0.9	50.0	18.7	81.3	1.1	0.4	2.3
May 20	0.1	0.0	0.4	62.5	24.5	91.5	0.8	0.4	1.5
Jun 20	0.0	0.0	0.3	14.3	0.4	57.9	1.8	1.1	2.7
Jul 20	0.0	0.0	0.6	41.9	24.5	60.9	2.0	1.1	3.5
Aug 20	0.0	0.0	0.6	30.0	6.7	65.2	1.2	0.5	2.5
Sep 20	0.2	0.0	0.8	25.0	0.6	80.6	1.4	0.7	2.4
Oct 20	0.2	0.0	0.7	0.0	0.0	41.0	1.8	1.1	2.8
Nov 20	0.1	0.0	0.5	0.0	0.0	70.8	1.0	0.5	1.8
Dec 20	0.0	0.0	0.4	100.0	47.8	100.0	1.8	1.1	2.9
Jan 21	0.1	0.0	0.4	50.0	11.8	88.2	1.0	0.5	1.7
Feb 21	0.0	0.0	0.3	33.3	0.8	90.6	1.2	0.7	2.0
Mar 21	0.0	0.0	0.6		0.0	100.0	1.7	0.8	3.1
Apr 21	0.0	0.0	7.4		0.0	100.0	2.1	0.1	11.1

Table S3. ASF virus prevalence of hunted wild boar including the 95% confidence intervals on municipality level in the years 2016 – 2021 (only the first 4 months for 2021). Blank fields indicate lack of data in the respective municipality.

	**2016**			**2017**			**2018**			**2019**			**2020**			**2021**		
District	**Prev in %**	**Lo CI in %**	**Up CI in %**	**Prev in %**	**Lo CI in %**	**Up CI in %**	**Prev in %**	**Lo CI in %**	**Up CI in %**	**Prev in %**	**Lo CI in %**	**Up CI in %**	**Prev in %**	**Lo CI in %**	**Up CI in %**	**Prev in %**	**Lo CI in %**	**Up CI in %**
Akmene	0	0	5.8	0	0	24.7	10.6	6.3	16.5	0.0	0.0	10.9	0.0	0.0	12.8	0.0	0.0	33.6
Alytus	0	0	0.7	0.9	0.2	2.2	2.6	1	5.5	1.6	0.2	5.7	0.0	0.0	2.1	0.0	0.0	4.7
Anyksciai	0.9	0.5	1.6	4.8	3.2	7.0	4	0.8	11.2	0.0	0.0	3.3	0.0	0.0	1.5	0.0	0.0	4.2
Birstonas	0	0	7.3	0	0	7.0	0	0	7.1	0.0	0.0	33.6	0.0	0.0	60.2		0.0	100.0
Birzai	0.9	0.3	2	3.7	1.9	6.6	3.6	0.7	10.1	0.0	0.0	3.9	0.0	0.0	1.9	0.0	0.0	4.1
Druskininkai	0	0	8	0	0	5.6	0	0	24.7	0.0	0.0	52.2	0.0	0.0	70.8	0.0	0.0	52.2
Elektrenai	0	0	0.8	0.5	0.1	1.7	3.3	1.6	6.1	0.0	0.0	4.1	0.0	0.0	3.3	0.0	0.0	12.8
Ignalina	4.4	1.6	9.4	0.9	0	5.0	0	0	2.4	2.2	0.5	6.4	1.1	0.1	4.0	0.0	0.0	5.7
Jonava	0.8	0.2	1.8	3.6	1.6	6.9	0	0	6.6	0.0	0.0	5.5	0.0	0.0	2.9	0.0	0.0	16.8
Joniskis	0	0	2.6	0	0	4.3	2.5	0.9	5.3	1.3	0.0	7.0	0.0	0.0	5.7	0.0	0.0	21.8
Jurbarkas	0	0	3.9	4.8	2.6	7.9	2.7	1.4	4.6	0.0	0.0	1.1	0.0	0.0	1.0	0.0	0.0	4.6
Kaisiadorys	0.2	0	0.9	0.5	0.1	1.4	1.8	0.8	3.3	2.5	1.1	4.9	0.4	0.0	2.2	0.0	0.0	7.5
Kalvarija	0	0	5.5	0	0	2.5	0	0	2	0.0	0.0	2.1	0.0	0.0	2.0	0.0	0.0	6.5
Kaunas	0.1	0	0.8	0.2	0	0.8	1	0.5	2	7.8	5.3	11.0	0.6	0.0	3.1	0.0	0.0	8.0
Kazlu Ruda	0	0	4.9	0	0	2.1	0	0	1.2	2.1	0.3	7.3	0.0	0.0	21.8	0.0	0.0	41.0
Kedainiai	0.2	0	0.5	2.2	1.5	2.9	1.9	1	3.5	0.0	0.0	1.1	0.0	0.0	1.0	0.0	0.0	4.2
Kelme	0	0	3.2	0	0	2.5	0.2	0	1	0.3	0.0	1.1	0.0	0.0	0.6	0.0	0.0	2.0
Klaipeda	0	0	6.4	0	0	10.0	0	0	11.2	0.0	0.0	2.1	0.0	0.0	0.9	0.0	0.0	3.1
Kretinga	0	0	3.7	0	0	4.1	0	0	5.3	0.0	0.0	5.1	0.0	0.0	1.9	0.0	0.0	2.7
Kupiskis	0	0	1.1	1.5	0.2	5.3	3.2	0.4	11	0.0	0.0	9.7	0.0	0.0	5.1	0.0	0.0	14.2
Lazdijai	0.2	0	1.2	2	0.9	3.7	3	1.2	6.1	1.7	0.2	6.1	0.0	0.0	3.5	0.0	0.0	13.2
Marijampole	0	0	3.2	0	0	1.6	0.3	0	1.8	1.6	0.2	5.6	0.0	0.0	8.6	0.0	0.0	33.6
Mazeikiai	0	0	4	0	0	6.1	0.6	0.1	1.7	3.1	1.6	5.5	2.5	0.5	7.1	0.0	0.0	18.5
Moletai	0.3	0	1.2	1.1	0.4	2.2	1	0.3	2.4	1.7	0.5	3.9	1.0	0.1	3.6	0.0	0.0	7.3
Neringa	0	0	21.8	0	0	97.5	0	0	11.2	0.0	0.0	3.5	0.0	0.0	26.5		0.0	100.0
Pagegiai	0	0	6	0	0	5.4	0	0	4.7	0.0	0.0	2.4	0.0	0.0	2.3	0.0	0.0	11.6
Pakruojis	0	0	11.9	3.6	1.3	7.6	6.8	3.7	11.3	0.0	0.0	5.3	0.0	0.0	6.3	0.0	0.0	17.6
Palanga	0	0	97.5	0	0	84.2	0	0	97.5	0.0	0.0	84.2		0.0	100.0		0.0	100.0

Panevezys	0.9	0.4	1.9	4.1	2.8	5.7	14.3	7.1	24.7	0.0	0.0	5.4	1.1	0.0	6.2	0.0	0.0	15.4
Pasvalys	0	0	2.9	4.8	2.3	8.6	0	0	4.5	0.0	0.0	5.1	0.0	0.0	3.7	0.0	0.0	8.8
Plunge	0	0	4.1	0	0	6.3	0	0	2	0.2	0.0	1.0	0.4	0.1	1.6	1.3	0.0	6.8
Prienai	0	0	0.5	0	0	0.5	1.3	0.6	2.5	2.8	0.9	6.5	0.0	0.0	3.7	0.0	0.0	26.5
Radviliskis	0	0	4.2	1.9	0.7	4.1	1.7	0.9	3.1	0.0	0.0	1.3	0.0	0.0	1.1	0.0	0.0	3.3
Raseiniai	0	0	3.9	0	0	2.2	0.8	0.2	2	0.0	0.0	1.0	0.0	0.0	1.3	0.0	0.0	3.7
Rietavas	0	0	5.8	0	0	11.9	0	0	4.8	0.0	0.0	1.6	0.0	0.0	1.1	0.0	0.0	5.6
Rokiskis	0.8	0.2	2	3.5	1.6	6.5	2.7	0.3	9.5	0.0	0.0	3.3	0.0	0.0	2.9	0.0	0.0	4.7
Sakiai	0	0	17.6	0	0	2.9	3.1	1.7	5.3	2.3	0.9	5.0	0.0	0.0	2.7	0.0	0.0	5.3
Salcininkai	0	0	1.6	1.3	0.2	4.7	0	0	2.8	0.7	0.0	3.8	0.0	0.0	2.3	0.0	0.0	9.7
Siauliai	0	0	3	0	0	2.8	2.5	1.1	4.9	1.3	0.3	3.7	0.0	0.0	1.4	0.0	0.0	4.3
Silale	0	0	5.6	0	0	9.7	0	0	1.4	0.0	0.0	0.8	0.0	0.0	0.9	0.0	0.0	2.7
Silute	0	0	7.5	0	0	16.1	0	0	3.1	0.0	0.0	1.3	0.0	0.0	0.9	0.0	0.0	3.2
Sirvintos	0	0	0.6	2.2	1.1	3.8	1.2	0	6.7	1.2	0.0	6.3	0.0	0.0	2.6	0.0	0.0	10.3
Skuodas	0	0	5.1	0	0	3.8	0	0	4.7	0.0	0.0	2.4	2.2	0.3	7.6	0.0	0.0	14.2
Svencionys	0	0	2.1	0.7	0	3.7	0.5	0	2.9	0.5	0.0	2.7	0.6	0.0	3.4	0.0	0.0	9.5
Taurage	0	0	4.5	0	0	8.8	0.4	0	2.3	0.0	0.0	0.8	0.0	0.0	0.8	0.0	0.0	3.2
Telsiai	0	0	3	0	0	2.9	0.8	0.3	1.7	1.1	0.5	2.1	0.4	0.0	1.3	0.0	0.0	1.8
Trakai	0	0	0.7	0.3	0	1.1	1.8	0.8	3.4	0.6	0.1	2.3	0.0	0.0	1.0	0.0	0.0	4.4
Ukmerge	0	0	0.5	2.5	1.5	3.7	5.3	1.5	12.9	0.0	0.0	6.3	0.0	0.0	3.0	0.0	0.0	9.5
Utena	0	0	0.5	1.3	0.5	2.5	1.4	0.4	3.5	0.6	0.0	3.3	0.0	0.0	2.9	0.0	0.0	14.2
Varena	0.3	0	1.7	1.6	0.5	3.6	2.2	0.6	5.4	0.0	0.0	3.0	0.0	0.0	1.3	0.0	0.0	5.9
Vilkaviskis	0	0	4.6	0.5	0	2.9	0	0	1.6	0.0	0.0	1.7	0.0	0.0	1.9	0.0	0.0	9.7
Vilnius	0.2	0	0.8	0.3	0	1.0	1.1	0.5	2.4	1.7	0.5	3.8	0.4	0.0	2.1	0.0	0.0	4.9
Visaginas		0	100	0	0	60.2		0	100		0.0	100.0		0.0	100.0	0.0	0.0	97.5
Zarasai	0.2	0	1.1	2.3	1	4.3	3.3	1.1	7.5	0.0	0.0	3.9	0.8	0.0	4.3	0.0	0.0	6.7

Table S4. ASF virus prevalence for wild boar found dead including the 95% confidence intervals on municipality level in the years 2016 - 2021 (only the first 4 months for 2021). Blank fields indicate lack of data in the respective municipality.

	**2016**			**2017**			**2018**			**2019**			**2020**			**2021**		
District	**Prev in %**	**Lo CI in %**	**Up CI in %**	**Prev in %**	**Lo CI in %**	**Up CI in %**	**Prev in %**	**Lo CI in %**	**Up CI in %**	**Prev in %**	**Lo CI in %**	**Up CI in %**	**Prev in %**	**Lo CI in %**	**Up CI in %**	**Prev in %**	**Lo CI in %**	**Up CI in %**
Akmene		0.0	100.0	100.0	2.5	100.0	76.7	66.4	85.2	0.0	0.0	97.5		0.0	100.0		0	100

Alytus	0.0	0.0	45.9	76.9	56.4	91.0	72.7	54.5	86.7	33.3	0.8	90.6	0.0	0.0	97.5		0	100
Anyksciai	79.3	69.6	87.1	89.2	82.8	93.8	25.0	0.6	80.6	0.0	0.0	84.2		0.0	100.0		0	100
Birstonas	0.0	0.0	97.5		0.0	100.0	100.0	2.5	100.0	50.0	1.3	98.7		0.0	100.0		0	100
Birzai	62.1	42.3	79.3	92.6	84.6	97.2	41.7	15.2	72.3		0.0	100.0	0.0	0.0	97.5		0	100
Druskininkai	0.0	0.0	97.5	75.0	19.4	99.4	0.0	0.0	97.5	50.0	1.3	98.7		0.0	100.0		0	100
Elektrenai	0.0	0.0	52.2	0.0	0.0	45.9	0.0	0.0	97.5		0.0	100.0	0.0	0.0	97.5		0	100
Ignalina	66.7	38.4	88.2	57.1	18.4	90.1	50.0	6.8	93.2	0.0	0.0	97.5	100.0	2.5	100.0	100.0	40.0	100
Jonava	70.8	60.7	79.7	82.9	67.9	92.8	40.0	5.3	85.3	0.0	0.0	52.2		0.0	100.0	0	0	98
Joniskis	0.0	0.0	97.5	0.0	0.0	60.2	40.0	16.3	67.7		0.0	100.0		0.0	100.0		0	100
Jurbarkas	0.0	0.0	33.6	62.7	49.1	75.0	76.7	69.4	83.1	0.0	0.0	52.2	0.0	0.0	97.5		0	100
Kaisiadorys	53.6	39.7	67.0	36.8	16.3	61.6	34.6	17.2	55.7	33.3	9.9	65.1	50.0	18.7	81.3		0	100
Kalvarija	0.0	0.0	97.5	0.0	0.0	70.8	0.0	0.0	84.2	0.0	0.0	97.5	0.0	0.0	97.5		0	100
Kaunas	79.2	68.5	87.6	0.0	0.0	20.6	71.8	55.1	85.0	56.5	45.3	67.2	55.6	35.3	74.5		0	100
Kazlu Ruda	0.0	0.0	52.2	0.0	0.0	52.2	0.0	0.0	52.2	25.0	0.6	80.6		0.0	100.0		0	100
Kedainiai	36.8	16.3	61.6	60.8	48.8	72.0	78.9	62.7	90.4	50.0	1.3	98.7		0.0	100.0		0	100
Kelme	0.0	0.0	60.2	0.0	0.0	97.5	50.0	1.3	98.7	33.3	0.8	90.6	0.0	0.0	84.2		0	100
Klaipeda	0.0	0.0	97.5		0.0	100.0	0.0	0.0	52.2	0.0	0.0	52.2		0.0	100.0		0	100
Kretinga		0.0	100.0	0.0	0.0	97.5	0.0	0.0	41.0	0.0	0.0	70.8	0.0	0.0	84.2		0	100
Kupiskis	60.0	14.7	94.7	75.0	34.9	96.8	28.6	3.7	71.0		0.0	100.0		0.0	100.0		0	100
Lazdijai	0.0	0.0	41.0	73.1	52.2	88.4	86.8	79.9	92.0	0.0	0.0	97.5	0.0	0.0	84.2		0	100
Marijampole	0.0	0.0	30.8	0.0	0.0	19.5	47.1	23.0	72.2	69.2	38.6	90.9		0.0	100.0		0	100
Mazeikiai	0.0	0.0	70.8	73.3	44.9	92.2	74.4	57.9	87.0	61.5	31.6	86.1	85.7	42.1	99.6		0	100
Moletai	0.0	0.0	21.8	71.1	54.1	84.6	87.5	47.3	99.7	0.0	0.0	84.2	75.0	34.9	96.8		0	100
Neringa		0.0	100.0		0.0	100.0		0.0	100.0	0.0	0.0	97.5		0.0	100.0		0	100
Pagegiai		0.0	100.0	0.0	0.0	70.8	0.0	0.0	84.2	0.0	0.0	60.2		0.0	100.0		0	100
Pakruojis		0.0	100.0	85.7	42.1	99.6	91.2	81.8	96.7	0.0	0.0	70.8	0.0	0.0	97.5		0	100
Palanga		0.0	100.0		0.0	100.0		0.0	100.0		0.0	100.0		0.0	100.0		0	100
Panevezys	60.9	38.5	80.3	89.3	80.1	95.3	0.0	0.0	97.5		0.0	100.0		0.0	100.0		0	100
Pasvalys	0.0	0.0	52.2	82.5	70.9	90.9	93.5	84.3	98.2	25.0	0.6	80.6	0.0	0.0	97.5		0	100
Plunge	0.0	0.0	97.5	0.0	0.0	97.5	0.0	0.0	70.8	0.0	0.0	84.2	47.6	25.7	70.2		0	100
Prienai	0.0	0.0	97.5	0.0	0.0	60.2	45.5	16.7	76.6	50.0	11.8	88.2		0.0	100.0		0	100
Radviliskis	0.0	0.0	84.2	0.0	0.0	36.9	74.5	59.7	86.1	33.3	0.8	90.6	0.0	0.0	97.5		0	100
Raseiniai	75.0	19.4	99.4	73.7	48.8	90.9	62.9	44.9	78.5	16.7	0.4	64.1	0.0	0.0	97.5		0	100
Rietavas	0.0	0.0	84.2	0.0	0.0	97.5		0.0	100.0	0.0	0.0	97.5		0.0	100.0		0	100
Rokiskis	0.0	0.0	70.8	80.0	61.4	92.3	91.7	61.5	99.8	0.0	0.0	97.5		0.0	100.0		0	100

Sakiai	0.0	0.0	97.5	0.0	0.0	84.2	100.0	29.2	100.0	94.4	72.7	99.9	0.0	0.0	84.2		0	100
Salcininkai	31.3	11.0	58.7	68.8	41.3	89.0	16.7	0.4	64.1	0.0	0.0	84.2	0.0	0.0	97.5		0	100
Siauliai	0.0	0.0	70.8	0.0	0.0	97.5	65.8	48.6	80.4	100.0	63.1	100.0	0.0	0.0	70.8		0	100
Silale	0.0	0.0	97.5	0.0	0.0	97.5	0.0	0.0	97.5	0.0	0.0	97.5	0.0	0.0	97.5		0	100
Silute	0.0	0.0	97.5		0.0	100.0	33.3	0.8	90.6	0.0	0.0	84.2		0.0	100.0	0	0	84
Sirvintos	0.0	0.0	36.9	45.8	25.6	67.2	77.1	62.7	88.0		0.0	100.0	0.0	0.0	84.2		0	100
Skuodas	0.0	0.0	84.2	0.0	0.0	70.8	0.0	0.0	84.2	50.0	1.3	98.7	0.0	0.0	84.2		0	100
Svencionys	0.0	0.0	97.5	0.0	0.0	97.5	33.3	0.8	90.6	75.0	34.9	96.8	62.5	35.4	84.8		0	100
Taurage	0.0	0.0	70.8	0.0	0.0	36.9	0.0	0.0	97.5	0.0	0.0	60.2	0.0	0.0	97.5		0	100
Telsiai	0.0	0.0	24.7	0.0	0.0	41.0	44.0	24.4	65.1	54.5	23.4	83.3	25.0	3.2	65.1		0	100
Trakai	25.0	0.6	80.6	20.0	0.5	71.6	66.7	9.4	99.2	0.0	0.0	84.2	0.0	0.0	97.5	0	0	84
Ukmerge	7.4	0.9	24.3	87.4	83.3	90.8	84.9	77.8	90.4	33.3	0.8	90.6	28.6	3.7	71.0		0	100
Utena	25.0	3.2	65.1	79.5	64.7	90.2	77.8	40.0	97.2	100.0	2.5	100.0		0.0	100.0		0	100
Varena	0.0	0.0	45.9	94.0	85.4	98.3	63.6	40.7	82.8		0.0	100.0		0.0	100.0		0	100
Vilkaviskis	0.0	0.0	70.8	0.0	0.0	60.2	0.0	0.0	36.9	0.0	0.0	60.2		0.0	100.0		0	100
Vilnius	4.5	0.1	22.8	7.1	0.2	33.9	45.5	24.4	67.8	60.0	26.2	87.8	66.7	9.4	99.2		0	100
Visaginas		0.0	100.0		0.0	100.0		0.0	100.0		0.0	100.0		0.0	100.0		0	100
Zarasai	62.5	24.5	91.5	78.7	66.3	88.1	88.9	73.9	96.9	33.3	0.8	90.6	33.3	0.8	90.6		0	100

Table S5. Seroprevalence in hunted wild boar including the 95% confidence intervals on municipality level in the years 2016 - 2021 (only the first 4 months for 2021). Blank fields indicate lack of data in the respective municipality.

	**2016**			**2017**			**2018**			**2019**			**2020**			**2021**		
District	**Prev in %**	**Lo CI in %**	**Up CI in %**	**Prev in %**	**Lo CI in %**	**Up CI in %**	**Prev in %**	**Lo CI in %**	**Up CI in %**	**Prev in %**	**Lo CI in %**	**Up CI in %**	**Prev in %**	**Lo CI in %**	**Up CI in %**	**Prev in %**	**Lo CI in %**	**Up CI in %**
Akmene	0	0	5.9	0	0	24.7	2.8	0.8	7	28.1	13.7	46.7	16.7	4.7	37.4	0.0	0.0	33.6
Alytus	0.2	0	1.1	0	0	0.8	2.6	1	5.5	1.6	0.2	5.8	0.0	0.0	2.1	0.0	0.0	4.7
Anyksciai	0.6	0.3	1.2	1.5	0.6	2.9	19.4	11.1	30.5	7.3	3.2	14.0	2.9	1.2	5.9	1.2	0.0	6.3
Birstonas	0	0	7.3	0	0	7	2	0.1	10.6	0.0	0.0	33.6	0.0	0.0	60.2		0.0	100.0
Birzai	1.2	0.5	2.5	2.8	1.2	5.4	3.7	0.8	10.3	4.4	1.2	11.0	2.1	0.6	5.2	0.0	0.0	4.1
Druskininkai	0	0	8.4	0	0	5.6	0	0	24.7	0.0	0.0	52.2	0.0	0.0	70.8	0.0	0.0	52.2
Elektrenai	0	0	0.8	0	0	0.9	1.4	0.4	3.4	1.1	0.0	6.2	0.9	0.0	5.0	0.0	0.0	12.8
Ignalina	2.3	0.5	6.6	0.9	0	5	2	0.4	5.8	2.3	0.5	6.5	0.6	0.0	3.1	0.0	0.0	5.7
Jonava	1.2	0.5	2.4	3.1	1.3	6.4	14.8	6.6	27.1	4.6	1.0	12.9	1.6	0.2	5.6	0.0	0.0	16.8

Joniskis	0	0	3	0	0	5.2	3.7	1.5	7.5	12.2	5.7	21.8	4.8	1.0	13.3	6.7	0.2	31.9
Jurbarkas	0	0	3.9	0.4	0	2	0.9	0.2	2.3	0.9	0.2	2.7	0.8	0.2	2.3	0.0	0.0	4.6
Kaisiadorys	0.4	0.1	1.1	0.8	0.3	1.9	2.2	1.1	3.8	0.9	0.2	2.7	1.2	0.3	3.5	2.1	0.1	11.3
Kalvarija	0	0	5.5	0	0	2.5	0	0	2	0.0	0.0	2.1	0.0	0.0	2.0	0.0	0.0	6.5
Kaunas	0.3	0	1.1	0.2	0	0.9	0.6	0.2	1.4	1.2	0.3	3.2	3.5	1.3	7.5	2.3	0.1	12.0
Kazlu Ruda	0	0	5.2	0	0	2.1	0	0	1.2	1.1	0.0	5.7	14.3	1.8	42.8	0.0	0.0	41.0
Kedainiai	0.2	0	0.5	0.6	0.3	1.1	2.3	1.2	3.9	4.3	2.4	7.2	0.9	0.2	2.5	2.4	0.3	8.2
Kelme	0	0	3.2	0	0	2.5	0	0	0.7	0.6	0.2	1.6	0.7	0.2	1.7	1.6	0.3	4.7
Klaipeda	0	0	10.6	0	0	10.6	0	0	20.6	0.0	0.0	2.2	0.2	0.0	1.3	0.8	0.0	4.6
Kretinga	0	0	5.4	0	0	4.2	0	0	5.4	0.0	0.0	5.3	0.5	0.0	2.9	0.0	0.0	2.7
Kupiskis	0.3	0	1.7	1.5	0.2	5.3	11.1	4.6	21.6	13.9	4.7	29.5	0.0	0.0	5.1	0.0	0.0	14.2
Lazdijai	0	0	0.8	0.2	0	1.2	0.9	0.1	3.1	1.7	0.2	6.1	0.0	0.0	3.5	0.0	0.0	13.2
Marijampole	0	0	3.2	0	0	1.6	0.3	0	1.8	0.0	0.0	3.0	0.0	0.0	8.8	0.0	0.0	33.6
Mazeikiai	0	0	4.1	0	0	6.2	0.8	0.2	2	3.2	1.6	5.7	1.7	0.2	6.0	5.6	0.1	27.3
Moletai	0.3	0	1.2	0	0	0.6	1.5	0.6	3.2	2.7	1.2	5.3	3.6	1.5	7.3	2.0	0.1	10.9
Neringa	0	0	97.5	0	0	97.5		0	100	0.0	0.0	3.9	0.0	0.0	26.5		0.0	100.0
Pagegiai	0	0	6	0	0	5.4	0	0	4.7	1.3	0.2	4.6	0.0	0.0	2.3	0.0	0.0	11.6
Pakruojis	0	0	11.9	0.6	0	3.3	4.2	1.8	8.1	0.0	0.0	5.3	1.8	0.0	9.6	5.3	0.1	26.0
Palanga		0	100	0	0	84.2	0	0	97.5	0.0	0.0	84.2		0.0	100.0		0.0	100.0
Panevezys	0	0	0.5	0.7	0.3	1.6	17.4	9.3	28.4	12.1	5.4	22.5	4.7	1.3	11.6	0.0	0.0	15.4
Pasvalys	2.4	0.5	7	1.9	0.5	4.9	10	4.4	18.8	4.3	0.9	12.0	2.1	0.3	7.3	0.0	0.0	8.8
Plunge	0	0	4.5	0	0	6.3	0	0	2.1	0.0	0.0	0.6	2.9	1.5	4.9	5.0	1.4	12.3
Prienai	0	0	0.5	0	0	0.5	0.6	0.2	1.5	2.3	0.6	5.8	5.2	1.7	11.6	0.0	0.0	26.5
Radviliskis	0	0	4.2	0.3	0	1.8	0.5	0.1	1.4	0.7	0.1	2.5	0.3	0.0	1.7	0.0	0.0	3.3
Raseiniai	0	0	4.1	0	0	2.3	0.4	0	1.4	0.0	0.0	1.1	1.1	0.2	3.1	0.0	0.0	3.7
Rietavas	1.7	0	9.1	0	0	28.5	0	0	5.8	0.5	0.0	2.5	0.0	0.0	1.1	1.6	0.0	8.4
Rokiskis	0.4	0	1.5	0	0	1.5	12.7	6	22.7	4.6	1.5	10.5	0.0	0.0	2.9	1.3	0.0	7.0
Sakiai	0	0	17.6	0.8	0	4.3	0	0	0.9	0.4	0.0	2.2	0.0	0.0	2.7	0.0	0.0	5.3
Salcininkai	2.6	1	5.6	2.2	0.5	6.4	0.8	0	4.2	2.1	0.4	6.1	0.6	0.0	3.5	2.8	0.1	14.5
Siauliai	0	0	3	0	0	2.8	4.4	2.4	7.3	9.5	6.0	14.0	1.2	0.2	3.4	9.6	4.3	18.1
Silale	0	0	5.9	0	0	10	0	0	1.4	0.2	0.0	1.2	0.0	0.0	0.9	0.7	0.0	4.0
Silute	0	0	8.2	0	0	16.1	0	0	3.2	0.4	0.0	2.0	0.0	0.0	0.9	0.0	0.0	3.2
Sirvintos	0	0	0.6	0.6	0.1	1.6	19.8	11.7	30.1	3.5	0.7	9.9	0.7	0.0	4.0	0.0	0.0	10.3
Skuodas	0	0	5.1	0	0	3.8	0	0	4.7	0.0	0.0	2.4	0.0	0.0	3.9	0.0	0.0	14.2
Svencionys	0	0	2.2	0	0	2.5	0	0	2.1	2.0	0.5	5.0	1.9	0.4	5.4	2.7	0.1	14.2

Taurage	0	0	4.5	0	0	8.8	0	0	1.5	0.0	0.0	0.8	0.2	0.0	1.3	0.9	0.0	4.8
Telsiai	0	0	3	0	0	2.9	0.2	0	0.9	1.6	0.9	2.9	2.4	1.3	4.0	1.4	0.3	4.2
Trakai	0.2	0	1.1	0.2	0	0.9	0.6	0.1	1.8	1.9	0.7	4.1	0.5	0.1	1.9	2.4	0.3	8.5
Ukmerge	0	0	0.5	0.9	0.4	1.8	16.2	8.7	26.6	12.5	5.2	24.1	7.8	3.6	14.2	2.7	0.1	14.2
Utena	0.1	0	0.8	0.6	0.2	1.6	2.1	0.8	4.5	4.8	2.1	9.2	6.8	3.0	12.9	0.0	0.0	14.2
Varena	1.3	0.4	3.3	0.6	0.1	2.3	3.3	1.2	7	2.5	0.5	7.1	1.1	0.2	3.2	0.0	0.0	5.9
Vilkaviskis	0	0	4.9	0	0	1.9	0	0	1.6	0.0	0.0	1.7	0.5	0.0	2.9	0.0	0.0	9.7
Vilnius	0.3	0	1.1	0.3	0	1	1.2	0.5	2.4	3.4	1.6	6.1	4.7	2.5	8.1	0.0	0.0	4.9
Visaginas		0	100	25	0.6	80.6		0	100		0.0	100.0		0.0	100.0	0.0	0.0	97.5
Zarasai	0	0	0.7	0.8	0.2	2.2	1.3	0.2	4.7	3.2	0.7	9.1	4.9	1.8	10.4	0.0	0.0	6.7

Table S6. Median of estimated number of wild boar/km² based on sightings and snow track per hunting year and the results of the Mann–Whitney U test. Significant differences between the hunting seasons are highlighted.

Hunting season	Median of estimated number of wild boar/km²	Hunting season					
		**2014/15**	**2015/16**	**2016/17**	**2017/18**	**2018/19**	**2019/20**
		P-value					
**2013/14**	0.33	1	1	1	0.32	0.01	0.04
**2014/15**	0.40		0.35	0.20	0.01	<0.001	<0.001
**2015/16**	0.28			1	1	0.02	0.15
**2016/17**	0.29				1	0.04	0.15
**2017/18**	0.20					1	1
**2018/19**	0.14						1
**2019/20**	0.17						

Table S7. Median of the estimated number of wild boar/km² based on hunting bag per year and the results of the Mann–Whitney U test. Significant differences between the hunting seasons are highlighted.

Year	Median of hunted number of wild boar/km²	Year			
		**2017**	**2018**	**2019**	**2020**
		P-value			
**2016**	0.53	1	0.07	<0.001	<0.001
**2017**	0.50		0.20	<0.001	<0.001
**2018**	0.25			0.26	0.01
**2019**	0.13				1
**2020**	0.09				
